# An IgE antibody targeting HER2 identified by clonal selection restricts breast cancer growth via immune-stimulating activities

**DOI:** 10.1186/s13046-025-03319-5

**Published:** 2025-02-12

**Authors:** Lais C. G. F. Palhares, Melanie Grandits, Katie Stoker, Jitesh Chauhan, Heng Sheng Sow, Gilbert O. Fruhwirth, Sophia Tsoka, James Birtley, Leanne Partington, Tim Wilson, Elizabeth Hardaker, Sophia N. Karagiannis, Heather J. Bax, Kevin FitzGerald

**Affiliations:** 1 St. John’s Institute of Dermatology, School of Basic & Medical Biosciences, & KHP Centre for Translational Medicine, King’s College London, London, SE1 9RT UK; 2 Epsilogen Ltd, Waterfront, ARC West London, Manbre Road, Hammersmith, London, W6 9RH UK; 3 Department of Informatics, Faculty of Natural, Mathematical and Engineering Sciences, King’s College London, Bush House, London, WC2B 4BG UK; 4 Department of Imaging Chemistry and Biology, School of Biomedical Engineering and Imaging Sciences, Kings’ College London, London, SE1 7EH UK; 5 Breast Cancer Now Research Unit, School of Cancer & Pharmaceutical Sciences, King’s College London, Guy’s Hospital, London, SE1 9RT UK

**Keywords:** Breast Cancer, HER2, IgE, Immunotherapy

## Abstract

**Background:**

Tumor-targeting IgE antibodies have elicited potent tumor-restricting effects by recruiting immune effector mechanisms. However, a dedicated platform for the generation, selection and evaluation of novel IgEs based on target antigen recognition and functional profiles has not been reported.

**Methods:**

By establishing an IgE class antibody therapeutic design platform to allow selection of lead candidates, we generated a panel of IgEs recognising the human epidermal growth factor receptor 2 (HER2), overexpressed in 15–20% of breast cancers. From 1840 phage display-generated variable region sequences panned against HER2, we engineered 30 full length IgE antibodies. We selected three clones based on biophysical properties, reactivity to HER2 + cancer cells, epitope reactivity and Fc-mediated anti-tumor profiles in vitro. Clones with cross-reactivity to rat HER2 were selected to allow functional evaluations in a fully immunocompetent syngeneic HER2 + rat breast cancer model.

**Results:**

IgE antibodies induced degranulation and antibody-dependent cellular cytotoxicity against human and rat HER2-expressing tumor cells in vitro. IgE antibody 26 demonstrated anti-tumor activity in a syngeneic HER2 + rat model, and a human HER2 + breast cancer xenograft model in mice reconstituted with human immune cells. Treatment was associated with enhanced immune cell infiltration and pro-inflammatory immune signatures, and downregulated cancer progression signaling pathways, in the tumor microenvironment.

**Conclusions:**

This study pioneers the design and generation of anti-HER2 IgE lead antibody candidates with immune-stimulating and tumor-restricting effects. The present work may pave the way for antibody engineering therapeutic opportunities for challenging-to-treat HER2-expressing cancers.

**Supplementary Information:**

The online version contains supplementary material available at 10.1186/s13046-025-03319-5.

## Background

The epidermal growth factor receptor 2 (HER2, also known as ERBB2), a member of the HER family of receptor tyrosine kinases, is overexpressed in 15–20% of primary breast cancers including 10% of estrogen receptor (ER)-positive breast cancers [1, 2]. HER2 expression is reported to correlate with tumor progression, aggressive disease and poorer patient outcomes through activation of oncogenic processes such as cell proliferation, migration, invasion, and angiogenesis. Based on these combined characteristics, HER2 is an established cancer cell-surface target for antibody targeted immune therapies [3, 4]. Traditionally, HER2 positive or negative status is clinically determined according to immunohistochemistry (IHC) and in situ hybridization (ISH) measurements of HER2 expression levels in tumor biopsies. While approximately 80% of breast cancers are classified as HER2 negative using the above criteria, more recent studies indicate that nearly 60% of these tumors are in fact HER2-low [5]. Currently, therapy options for HER2-medium/low breast cancer remain limited [6, 7]. Monoclonal anti-HER2 antibodies, such as Trastuzumab and Pertuzumab (both antibodies of the immunoglobulin G (IgG) isotype) offer significant clinical benefit. However, multiple mechanisms of resistance to their Fab-mediated effects translate to a significant proportion of patients displaying inherent or acquired resistance to these therapies [8, 9]. Although anti-HER2 antibodies possess an active IgG1 Fc region, the contribution of the Fc domains of these antibodies may be restricted by impaired immune responses. These include immunosuppressive macrophages, exhausted NK cells and expression of inhibitory FcγRs by effector cells in the tumor microenvironment (TME). Therefore, novel antibody formats capable of differentially engaging effector cells in the breast cancer TME are desirable.

All clinically-available monoclonal antibody therapies, including those targeting HER2, are of the IgG class. Immunoglobulin E (IgE) antibodies may offer therapeutic advantages over IgGs based on several attributes which may allow antibodies of this class to operate in immune privileged sites such as tumors. These include very high affinity for the cognate high-affinity Fcε receptor (FcεRI), which is 100–10,000-fold greater than the affinity of IgGs for their Fcγ receptors, as well as the lack of inhibitory Fcε receptors. High affinity translates to slow dissociation of IgE from FcεRI on effector cells and this may result in more optimal immune activation signals, longer tissue residence, and immune surveillance within tumors [10–12]. MOv18 IgE, the first IgE antibody to enter the clinic, targets the cancer-associated antigen Folate Receptor-alpha (FRα), known to be overexpressed in several cancers including ovarian, endometrial, Triple-negative breast cancer and lung [13–17]. MOv18 IgE has shown a good safety profile and early signs of efficacy in late-stage ovarian cancer patients in a Phase I clinical trial (NCT02546921) [18]. Several IgE monoclonal antibodies specific for different cancer-associated antigens have shown potent in vitro and in vivo anti-tumor functions [18–20]. These antibodies have been shown to modulate the TME, by activating immune cells and stimulating pro-inflammatory signalling pathways, chemokines and cytokines [19–21]. Together, these highlight the promise of this antibody class to potentiate the recruitment and activation of a patient’s immune system, and the modulation of the TME through immune mechanisms not normally accessed by IgG. Previous studies have reported the anti-tumor functions for trastuzumab-equivalent anti-HER2 IgE [11, 22, 23]. However, the de novo discovery of IgEs based on target binding and isotype functionality, enabling a systematic assessment of tumor-restricting activity and pro-inflammatory mechanisms, has not been conducted.

In this study, we introduce a platform for the discovery, design, generation and functional evaluation of anti-HER2 IgE antibody therapeutic candidates. We employed phage display to generate 1840 antibody variable region domains reactive to HER2. Based on target binding and specificity characteristics, we selected 30 clones which were generated as full length anti-HER2 IgEs. From these, we selected three full-length IgE antibodies according to acceptable biophysical attributes, HER2 + cancer cell binding, epitope reactivity and in vitro anti-tumoral activity. In particular, rat HER2 cross-reactivity was built into the phage display and in vitro selection campaign to facilitate functional evaluations in a fully immunocompetent syngeneic rat model of a medium/low rHER2-expressing breast cancer. The selection of the rat as an appropriate in vivo model system was based on comparable FcεR expression, and immune cell distribution, between humans and rats [24–26]. We engineered the three selected anti-HER2 IgEs with rat Fc epsilon (Fcε) constant domains and assessed Fc-mediated effector functions against rHER2-expressing cancer cells in vitro. We then evaluated these three IgEs in the aforementioned rat in vivo model. We investigated treatment-associated tumor growth restriction, and immune cell infiltration, alongside modulation of immune and cancer progression signalling pathways, in tumors. The corresponding human IgE was also evaluated in vivo using an immunodeficient mouse model administered with human immune cells to serve as effectors. This medium/low (Herceptest 2 +) hHER2-expressing breast cancer xenograft model is resistant to Trastuzumab direct effects [8, 9] and allowed the study of only Fc-mediated efficacy of our IgE.

## Materials and methods

### Phage display

Antibody selection using phage display was performed by Iontas Ltd. The library contained VH and VL repertoires that were generated via rounds of PCR amplification from human peripheral blood mononuclear cells (PBMCs) isolated from 42 donors, and tonsil cells obtained from one donor. These VH and VL sequences were then assembled into a final scFv format that was fused to the G3P coat protein of the phage. Sequential phage library screening with panning to select for binding to human HER2 (hHER2), no binding to human HER1, followed by binding to rat HER2 (rHER2) was then undertaken [27–29].

### IgE antibody production and biophysical characterisation

HER2-targeted IgE antibody variants were produced in stably transfected CHO-K1 cells (RRID:CVCL_0214) and 4-hydroxy-3-nitro-phenacetyl (NIP) IgE specific for the hapten NIP was produced in Expi293F cells (RRID:CVCL_D615) as previously described [30]. All antibodies were purified using a CaptureSelect™ IgE column followed by a buffer exchange to PBS 7.4. Affinity purified samples were further purified by Size Exclusion-High Performance Liquid Chromatography (SEC-HPLC) using a Zorbax GF-250 9.4 mm ID x 25 cm column (Agilent) on an Agilent 1200 series HPLC system.

Samples for Sodium Dodecyl Sulfate–Polyacrylamide Gel Electrophoresis (SDS-PAGE) were prepared using BioRad 4 × Sample buffer with/without 1 μL Mercaptoethanol added (for reducing or non-reducing conditions, respectively). Samples were heated at 95 °C for 5 min and then loaded onto a 4–20% Mini-PROTEAN® TGX™ Precast (Bio-Rad). The gel was run at constant voltage of 120 V for 1 h in 1 × Tris–Glycine SDS buffer. Staining was performed using InstantBlue® Coomassie Protein Stain.

### hHER2 and rHER2 ELISAs

Human and rat HER2 reactivity of scFv and IgE antibodies were assessed by ELISA. MaxiSORP plates (Nunc) were coated with 50 μl/well of 3 μg/ml recombinant hHER2 or rHER2 (Sino Biologicals Cat# 10,004-H08H4 and Cat# 80,079-R08H). Following incubation at 4 °C overnight, 250 μl/well SuperBlockTM (Perbio Science Ltd.) was added for 2 h at room temperature (RT). Hexa-His-tagged scFv, or IgE antibodies, were added 50 μl/ well, and incubated for 2 h at RT. Following 4 × wash with PBS-0.05% Tween® 20 anti-His- or anti-human IgE-HRP detection antibodies were added and incubated for 30 min at RT. Plates were developed with OPD (Sigma) diluted in stable peroxidase substrate buffer (Pierce), for 5–10 min at RT, in darkness, followed by 50 μl/well 1 M HCl solution (Sigma). A Fluostar Omega microplate reader (BMG LABTECH) was used with an absorbance of 492 nm, and correction wavelength of 650 nm.

### Surface plasmon resonance (SPR)

Binding kinetics of human and rat anti-HER2 IgE antibodies to human FcεRI and human HER2 were evaluated by SPR using a Biacore T200 at 25 °C and analysed using Biacore T200 Evaluation Software V 2.0.1.

For human FcεRI binding, a CM5 chip was first coupled with ~ 9000 RU anti-His antibody (Cytiva Cat# 27–4710-01, RRID:AB_771435) using standard amine chemistry. His-tagged human FcεRI (R&D Systems) ligand, diluted 0.25 μg/ml in HBS-P + running buffer) was applied to ~ 60 RU at 10 μl/min. A five point three-fold dilution of anti-HER2 human or rat IgE antibody analyte (titrated 0.411 nM to 33.33 nM) was then applied. Flow rate was 30 μl/min, association time 200 s, dissociation time 600 s. Regeneration was performed with 2 × injections of 10 mM glycine pH 1.5.

For human HER2 binding of human IgE antibodies, a CM5 chip was coupled with ~ 9000 RU of a mixture of anti-kappa and anti-lambda monoclonal antibody using standard amine chemistry (Fab capture kit; Cytiva CAT# 28,958,325). Anti-HER2 human IgE antibody ligand, diluted to 10 µg/mL in running buffer, was captured to ~ 45 RU at 10 μl/min. 2 × 4 point twofold dilution of human HER2 (Sino Biological) analyte (titrated 160 to 20 nM and 10 to 1.25 nM) was then applied. Flow rate was 40 μl/min, association time 240 s, dissociation time 900 s. Regeneration was performed with 2 × injections of 10 mM glycine pH 2.1.

For human HER2 binding of rat IgE antibodies, a CM5 chip was coupled with ~ 9000 RU of anti-Mouse IgG (Cytiva) using standard amine chemistry. Mouse anti-Rat IgE secondary antibody (Thermo Fisher Scientific Cat# MA5-16,810, RRID:AB_2538293), diluted in running buffer to 10 µg/ml, was then captured to ~ 700 RU at 10 µl/min. Anti-HER2 rat IgE antibody, diluted in running buffer to ~ 10 µg/mL, was then captured to ~ 300 RU at 10 μl/min. A five point two-fold dilution of human HER2 (Sino Biologicals) analyte (titrated from 10 to 160 nM) was then applied. Flow rate 30 μl/min, association time 240 s, dissociation time 600 s. Regeneration was performed with 2 × injections of 10 mM glycine pH 1.7.

### Cell culture and PBMC isolation

All cell lines were maintained at 5% CO_2_ and 37 °C. RPMI1640 and DMEM were supplemented with 10% FCS, penicillin (5,000 U/ml), streptomycin (100 μg/ml). Human HER2 expressing breast cancer cell lines SKBR3 (ATCC HTB-30, RRID:CVCL_0033) and JIMT-1 (DSMZ ACC 589, RRID:CVCL_2077) were cultured in complete DMEM. Rat mammary adenocarcinoma cell line MTLn3 (kindly provided by Prof. Jeffrey E Segall, Albert Einstein College of Medicine) was cultivated in MEM media supplemented with 5% FCS. The human monocytic cell line U937 (ATCC CRL-1593.2, RRID:CVCL_0007) and the rat basophilic cell line RBL-SX38, which express both the native rat FcεRI and the human tetrameric form of FcεRI, (kindly provided by Prof. Jean-Pierre Kinet) were both cultured using complete RPMI, with the latter grown with additional G418 (Gibco/Thermo Fisher Scientific) antibiotic selection. All cell lines were periodically tested for mycoplasma contamination. Peripheral blood mononuclear cells (PBMCs) were freshly isolated from whole blood of healthy volunteers (individuals of any age, male or female, with no history of significant health problems) and of Fischer F344 rats (Janvier) using Ficoll-Paque, as previously described [19].

### In vitro and ex vivo assays

#### HER2 and FcεR cell-surface binding of anti-HER2 IgE antibodies

As previously described [19], 1 × 10^5^ RBL-SX38 cells, JIMT-1, and SKBR3 human breast cancer cells, and HH-16.cl-4, CC531 and MTLn3 rat cancer cells were incubated with anti-HER2 IgE antibodies or IgE isotype control at 5 µg/ml for 30 min at 4 °C, followed by a wash with FACS buffer (PBS pH 7.2 + 2% FBS). Next, 20 µg/ml of anti-human IgE-FITC (Vector Laboratories Cat# FI-3040, RRID:AB_2336145) or anti-rat IgE-FITC (Thermo Fisher Scientific Cat# SA1-25,261, RRID:AB_794561) detection antibody was added, and cells were incubated for another 30 min at 4 °C and washed twice with FACS buffer before acquisition using a flow cytometer (Fortessa, Becton Dickinson).

#### Epitope competition

To assess the HER2 epitope reactivity of IgE antibodies, 1 × 10^5^ SKBR3 cells were incubated with 5 µg/ml anti-HER2 IgE or IgE isotype control for 15 min at 4 °C. No IgE antibody control conditions were included. Followed by 2 × wash with FACS buffer, Trastuzumab and Pertuzumab IgG antibodies were added at 4 µg/ml. Following incubation at 4 °C for 15 min, the cells were washed 2 × with FACS buffer and then incubated with 20 µg/ml anti-human IgG-FITC detection antibody (Thermo Fisher Scientific Cat# H10301, RRID:AB_2536550) for 15 min at 4 °C. Cells were finally washed 2 × with FACS buffer, and then acquired using a flow cytometer (Fortessa, Becton Dickinson). Reduced MFI, as compared to control conditions (no IgE antibody + IgG antibody + anti-human IgG-FITC detection), was indicative of blockade of Trastuzumab or Pertuzumab IgG binding epitope by anti-HER2 IgE antibodies.

#### Degranulation assay

As previously described [19], RBL-SX38 cells were seeded at 1 × 10^4^ cells/well in a flat bottom 96-well plate in complete RPMI and incubated overnight at 37 °C, 5% CO_2_. Antibodies were added at a final concentration of 1.05 nM and the plate was incubated for 1 h. The supernatant was removed and three washes with HBSS-1%BSA (heat shock fraction) were performed before adding 3 × 10^4^ target tumor cells resuspended in HBSS-1%BSA. Plates were incubated for 30 min and supernatant then carefully removed. 20 ul supernatant were diluted with 30 ul HBSS-1%BSA buffer in black flat bottom 96-well plates and 50 µl β-hexosaminidase substrate (4-Methylumbelliferyl N-acetyl-β-D-glucosaminide) added. Plates were incubated for 2 h at 37 °C. The reaction was stopped by addition of 100 µl 0.5 M Tris–HCl buffer, and fluorescence was measured at 350 nm excitation and 450 nm emission using a Fluostar Omega microplate reader (BMG Labtech).

#### Three-color flow cytometric ADCC/ADCP tumor cell killing assay

As previously described [19, 24, 31] tumor cells were stained one day prior to assay with Carboxyfluorescein Succinimidyl Ester (CFSE) (Molecular Probes (Life Technologies)). Briefly, tumor cells were detached with 0.5 M EDTA for up to 10 min, washed by centrifugation (1200 rpm for 5 min) in standard culture media and then in serum-free Hank’s balanced salt solution (HBSS, Life Technologies). 0.75 μl of 0.5 μM CFSE/ 1 × 10^6^ tumor cells were incubated for 10 min at 37 °C. Cells were washed with complete media and centrifuged as above for 5 min. The cell pellet was resuspended in complete media and returned to culture overnight. The following day, CFSE labelled cells were detached, washed, counted and resuspended in complete culture media to 1 × 10^6^ cells/ml. 100 μl human or rat effector cells and 100 μl tumor cells (Effector:Target ratio of 3:1 for U937 and human cancer cells, 5:1 for RBL-SX38 and rat cancer cells, and 10:1 for human or rat PBMCs with human or rat cancer cells, respectively) co-incubated, together with antibodies as follows. Control samples were incubated with either no antibody or with 5 μg/ml non-specific isotype control IgE. Test samples were incubated with 5 μg/ml anti-HER2 IgE antibodies. After incubation for 3 h at 37 °C/5% CO2, cells were washed with FACS buffer (PBS supplemented with 5% BSA) and incubated for 20 min at 4 °C with 2 μg/ml APC-conjugated anti-human (BD Biosciences Cat# 561,864, RRID:AB_11153499) or anti-rat CD45 (BioLegend Cat# 202,221, RRID:AB_2632871) antibody to label human or rat immune cells, respectively. Cells were washed and resuspended in FACS buffer. Then dead cells were labelled for 2 min at 4 °C with DAPI (1:10,000, Life Technologies) and samples were immediately acquired on a flow cytometer (Fortessa, Becton Dickinson). Analysis was performed using FlowJo version 10.7.1 (Becton Dickinson, (RRID:SCR_008520). Flow plots, gating strategy, and percentage of antibody-dependent cell-mediated cytotoxicity (ADCC) and antibody-dependent cell-mediated phagocytosis (ADCP) were applied and calculated as per previous reports [19, 24, 31].

#### Fab-mediated direct effects

pHER2 inhibition of HER2 IgEs was assessed using the CisBio Phospho-HER2 (Tyr1221/1222) and Total HER2 cellular detection kit (Cisbio Bioassays Cat# 64HR2TDA), respectively, according to manufacturer’s instructions. Briefly, 5 × 10^4^/well cells were seeded in a 96-well flat-bottom plate and incubated overnight (5% CO_2_, 37 °C). Complete media was then replaced with 50 µl of serum-free media and incubated for 2 h before treatment with anti-HER2 IgE antibodies or isotype control at 5 µg/mL for 30 min, followed by addition of 50 µl 0.15 µg/ml hEGF for a further 10 min (incubations at 37 °C, 5% CO_2_). Cells were lysed with 50 µl of lysing buffer for 30 min at room temperature, and under gentle agitation. 16 µl of the lysate were transferred into a HTRF 96-well low volume plate containing 4 µl PhosphoHER2, or Total HER2, premixed with Eu-cryptate antibodies, provided in the kit. After 4 h incubation at room temperature, the HTRF signal was measured (excitation 337 nm, emission 620 nm (donor) and 665 nm (acceptor)). Levels of HER2 phosphorylation were calculated as a ratio of of HER2 phosphorylation/ total HER2.

For colony formation assay, MTLn3 cells (100 cells/well) were seeded in a 6-well flat-bottomed plate and cultured in complete media at 37 °C and 5% CO_2_. After 4 h, IgE isotype control or anti-HER2 IgE antibodies were added at the 5 μg/mL. The cells were washed with PBS solution every 3 days and the media renewed with fresh complete media containing the antibodies at the same concentrations. After a total of 10 days incubation time, the media was removed, and the cells washed, fixed with methanol and stained with 0.2% crystal violet, for 30 min. The excess stain was washed with PBS and the plate was air dried. Colony formation was determined by quantification of the crystal violet staining: stained colonies were solubilised with 10% acetic acid and 100 µl of the content of each well were transferred to a 96-well flat bottom plate and absorbance was determined at 570 nm using a Fluostar Omega microplate reader (BMG Labtech). Values were expressed as the percentage of the area in relation to the no antibody control.

The effect of anti-HER2 IgE antibodies on MTLn3 cell migration was investigated using Transwell® motility inserts in a 24-well plate. A cylindrical 8 μm pore size insert was placed inside each well. Each insert was initially hydrated by the addition of 500μL of serum-free MEM and tumor cells (3 × 10^5^) were resuspended in 300 μl of serum-free media and added to the upper compartment of inserts for adhesion. After 1 h, 50 μl of complete media alone (control without antibody), or containing the test IgE antibody at 5 μg/ml, were added to cells. Cell migration was stimulated by the addition of 500 μl of 30 μg/ml of fibronectin in complete MEM to the bottom compartment of Transwell® inserts. Wells containing only complete MEM were used as negative control for migration. Following incubation at 37 °C and 5% CO_2_ for 4 h, cells on the upper surface were carefully removed using a cotton-tipped applicator. Cells attached to the underside of the membrane represented the migrated cells. The inserts were then washed with PBS and cells were fixed with 10% methanol and stained with 0.2% crystal violet for 30 min. Images were captured using an inverted microscope (Nikon, TE-300). Next, 10% acetic acid was added to the inserts and incubated at RT for 20 min in agitation. Solubilised crystal violet-stained cells were measured by Fluostar Omega microplate reader (BMG Labtech) (570 nm excitation, 630 nm emission), and the number of migrated cells quantified as the percentage of the fibronectin-containing, no antibody control.

Cell viability was assessed by seeding 1 × 10^3^ JIMT-1 cells/well in a clear flat-bottom 96-well plate in serum-reduced (1% FBS) media. Following incubation overnight at 37 °C and 5% CO_2_, IgE antibodies were added at the indicated concentrations. At day 5, 100 µl of 10% MTS reagent (diluted in media) was added to the wells. After 2 h of incubation at 37 °C for 2 h, the plate measured by Fluostar Omega microplate reader (BMG Labtech) (490 nm excitation, 650 nm emission. Cell viability was expressed as a percentage of no antibody control.

#### In vivo models of HER2 expressing breast cancer

##### Subcutaneous MTLn3 rat syngeneic immunocompetent model

3–4 weeks-old female Fischer F344 rats (F344/HanZtmRj, Janvier, RRID:RGD_737893) were maintained and handled in accordance with the Institutional Committees on Animal Welfare of the UK Home Office (The Home Office Animals Scientific Procedures Act, 1986). Rats were subcutaneously (*s.c.*) injected with 0.7 × 10^6^ MTLn3 cancer cells next to the mammary fat pad (day 0). Once the tumors became measurable (day 12 after tumor challenge), animals were equally allocated to treatment groups.

For the anti-HER2 IgE antibody efficacy study, on day 13, animals were injected intravenously (*i.v.*) with 20 mg/kg of rat IgE, or PBS as a vehicle control, twice-weekly for a maximum of 5 doses. Animals remained in the study until tumor dimensions approached the limits set by Animal Welfare guidelines (up to 47 days after tumor challenge), when they were humanly euthanised. Tumors were harvested, halved and then stored in 10% buffered formalin or RNAlater for further histologic and bulk-transcriptomics analysis, respectively.

For the dose and schedule study, on day 13 animals were injected *i.v.* with 10 mg/kg or 20 mg/kg rat IgE or PBS on weekly or twice-weekly schedules for 2 weeks (total of 2 and 4 doses for the weekly and bi-weekly groups, respectively).

The comparison study of the efficacy of rat IgE, compared to a rat IgE isotype control was performed with 7 mg/kg *i.v.* IgE injections on a twice-weekly dosing schedule starting on day 12. Animals were humanly euthanised on day 25.

Animals were daily monitored, and special attention was directed to detecting any adverse events in this model. Any animal showing distress or pain reaching a moderate severity limit (sensitivity to handling, piloerection or persistent hunched posture), or any clinical signs suggestive of tumor growth reaching the above criteria, were humanely killed. Animals were humanly euthanised on day 25 or 26 or if presenting a tumor with a mean diameter of 2.8 cm. Weight loss of 15% in the presence of other clinical signs above or weight loss of 20% in the absence of any other clinical signs would also result in humane killing.

Tumor growth was monitored and measured with callipers 3 × weekly. Tumor size (mm^3^) was calculated using the following formula: mm^3^ = π x D x d^2^ / 6 (d = smallest diameter of tumor; D = largest diameter of tumor). Animals were monitored for 2 h post injection and adverse events recorded.

##### Subcutaneous JIMT-1 xenograft model in immunodeficient mice reconstituted with human PBMC

For the dose and scheduling study of human IgE 26, 6–8 weeks-old NOD-*Prkdc*^*scid*^*-IL2rg*^*Tm1*^/Rj (NXG, Janvier) female mice were injected *s.c.* into the right flank with 1 × 10^7^ JIMT-1 breast cancer cells 1:1 in Matrigel® (day 0). Once tumors measured approximately 50 mm^3^ (12 days post-inoculation), 5 × 10^6^ PBMCs together with 2, 10 or 20 mg/kg human IgE 26, or PBS control, were injected *i.v.*. Subsequent antibody injections (without PBMCs) were administrated on a fortnightly (Q14D), weekly (QW) or bi-weekly (BIW) schedule. Flt3 ligand (for myeloid cell boost) was administered at 10 μg/mouse on days 12, 13 and 14. Animals were humanly euthanised on day 40 day. A comparative study evaluating the efficacy of human IgE versus a human IgE isotype control, was performed with 20 mg/kg *i.v.* IgE injections on a twice-weekly dosing schedule.

The impact of human PBMC effector cells on human IgE 26 efficacy was assessed by a comparison study of 20 mg/kg *i.v.* IgE injections on a twice-weekly dosing schedule, together with, or without 5 × 10^6^ human PBMCs in the first dose.

Any animal showing distress or pain reaching a moderate severity limit (sensitivity to handling, piloerection or persistent hunched posture), or any clinical signs suggestive of tumour growth reaching the above criteria, were humanely killed. Animals were humanly euthanised on day 40 or if reaching a tumor volume of 1700 mm^3^. Weight loss of 20% would also result in humane killing.

Tumor growth was measured with callipers 3 × weekly, and tumor size (mm^3^) was calculated using the formula: mm^3^ = d2 x (D/2) (d = smallest diameter of tumor; D = largest diameter of tumor).

In all in vivo studies, tumor volume was measured and calculated blindly, without prior knowledge of the animal groups tested. Animal group size was calculated using power analysis.

#### Retention of rat HER2 expression in vivo

Following in vivo MTLn3 rat studies, retention of rat HER2 expression on tumors was evaluated by flow cytometry. Tumor samples were digested by incubation in complete RPMI media with 2 mg/mL Collagenase II (Life Technologies, Cat# 17,101–015) and 50 U/ml nuclease (Thermo, Cat# 88,700, 1:500) at 37 °C. After 45 min of incubation, samples were washed with serum-free RPMI, filtered and resuspended in FACS buffer. After centrifugation for 10 min at 600 g at 4 °C, sample pellets were resuspended in FACS buffer and stained for rat immune cell marker CD45, rat epithelial cell marker EpCAM and rat HER2 antibodies (anti-rat CD45 APC-conjugated, clone OX-1 (BioLegend Cat# 202,221, RRID:AB_2632871); anti-rat EpCAM Alexa fluor 594-conjugated (BioLegend Cat# 118,222, RRID:AB_2563322) and anti-rat HER2 FITC-conjugated (Novus Cat# NBP2-34641AF488, RRID:AB_3290225) antibodies, respectively). Samples were acquired on a flow cytometer (Fortessa, Becton Dickinson). Tumor retention of rat HER2 expression was also investigated in excised tumors by immunohistochemical analysis using anti-rat HER2 antibody (ab237715, Abcam). Detection was achieved by using a Bond® Polymer Refine Detection kit, containing a universal (IgG anti-rabbit/mouse) HRP-conjugated polymer (Leica Biosystems), followed by the application of DAB chromogenic substrate (DAKO), following the manufacturer’s instructions. This was followed by Mayer’s haematoxylin solution counterstaining (Sigma Aldrich).

#### Immunohistochemical analyses of MTLn3 tumor samples

Harvested tumors were sliced into sections and immune cell infiltration was investigated by immunohistochemistry. Anti-rat CD68 (Abcam Cat# ab125212, RRID:AB_10975465), CD3 (Abcam Cat# ab5690, RRID:AB_305055), CD4 (Cell Signaling Technology Cat# 25,229, RRID:AB_2798898) and CD8 (Abcam Cat# ab33786, RRID:AB_726709) antibodies were used to detect the respective markers, with HRP-conjugated horse anti-rabbit IgG polymer detection kit peroxidase (Vector Laboratories Cat# MP-7401, RRID:AB_2336529), following the manufacturer’s instructions. This was followed by Mayer’s haematoxylin solution counterstaining (Sigma Aldrich). The stained samples were digitally scanned at 20 × using the Hamamatsu NanoZoomer S360 whole slide scanner. Quantitative Image analysis was performed using the Visiopharm® (Version 2023.01.2.13695) Image Analysis Platform.

#### Transcriptomic analysis of MTLn3 tumors samples

Samples of MTLn3 tumors harvested at the end of the efficacy study underwent transcriptomics analysis (PBS, *n* = 6; rat IgE 20, *n* = 6; rat IgE 23, *n* = 6; and rat IgE 26 *n* = 7). RNA extraction of the samples was followed by Bulk RNA-sequencing using Illumina NovaSeq 2 × 150 bp sequencing, 350 M read pairs (Illumina, Ambion). All data analysis was conducted with R version 4.3.1 (2023–06–16). Rat genes were converted to human using the Babelgene (22.9) library tool. Differentially expressed genes (DEGs) between treatment and PBS control tumors were identified using the package DESeq2 (1.40.2) (RRID:SCR_015687).

Immune signature expression analysis and deconvolution was performed using TPM-normalised data and the ConsensusTME (0.0.1.9000) library with cancer type defined as ‘BRCA’ and single sample GSEA (ssGSEA) chosen as statistical method. For gene set enrichment analysis (GSEA) genes were ranked according to fold change and normalised enrichment score (NES) was calculated using package fgsea (1.26.0) with random seed set to 13, with Reactome (7.4) used as the chosen database. Gene sets of interest were visualized using ggplot2 (3.5.1) (RRID:SCR_014601). Code information can be found on https://github.com/katiestoker/palhares.git.

### Statistical analyses

All statistical analyses were performed using GraphPad™ Prism software (version 10.2.1 (339) (RRID:SCR_002798). *P* values are represented as follows: * = *p* < 0.05, ** = *p* < 0.01, *** = *p* < 0.001, **** = *p* < 0.0001. Error bars represent SD (Standard Deviation) and SEM (Standard Error of the Mean) for in vitro analyses, and in in vivo and histological analyses, respectively. A Shapiro–Wilk normality test was performed to evaluate normality of the data, and the most appropriate statistical analysis to compare data between experimental conditions was then selected. Details of the statistical tests applied are included in the figure legends.

## Results

### Phage display screening, generation and evaluation of anti-HER2 IgEs for antigen specificity, cross-reactivity to rat HER2, and in vitro Fc-mediated function

**Fig. 1:**
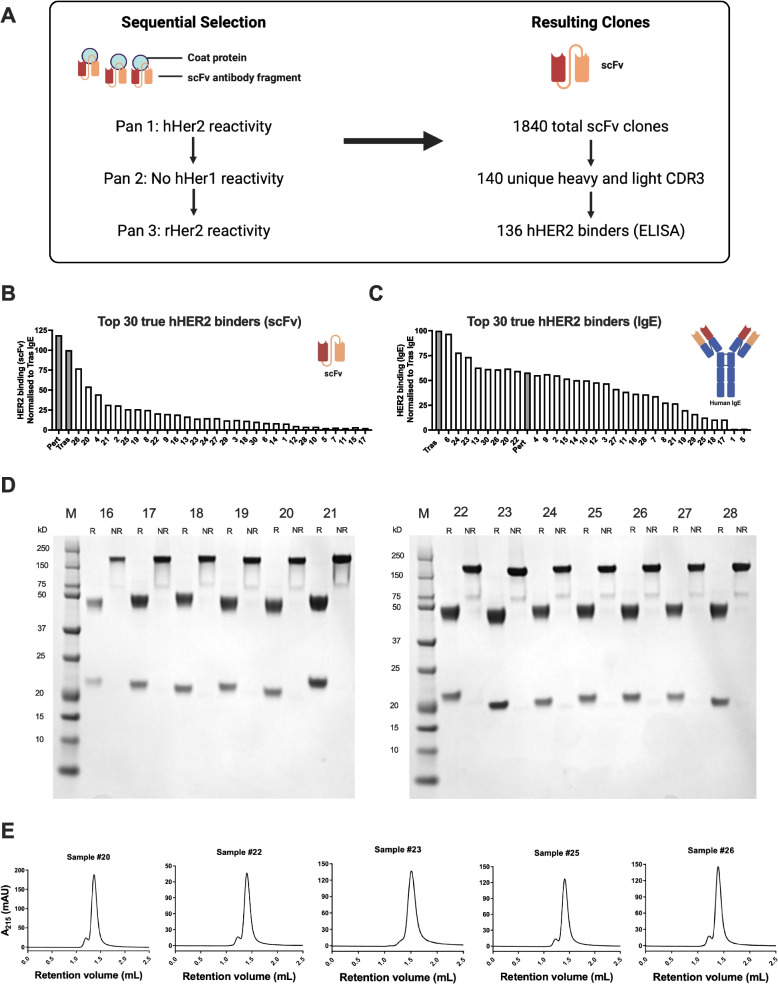
Phage display screening for HER2-binding scFvs, generation, and biophysical characterization of anti-HER2 IgE antibodies. **A**. Phage display approach, with sequential panning for binding to human HER2 (hHER2), but not human HER1, followed by binding to rat HER2 (rHER2), resulted in 1840 scFv constructs of which 140 had unique heavy and light chain CDR3 sequences and 136 were confirmed to bind hHER2 by ELISA. **B-C**. hHER2 binding was confirmed by ELISA for the 30 highest binding scFv constructs (**B**) and re-evaluated following production of these as full-length human IgE antibodies (**C**). **D-E**. SDS-PAGE (reduced (left lanes) and non-reduced (right lanes) conditions) (**D**) and size-exclusion HPLC (**E**) analyses confirmed the expected size and purity of anti-HER2 IgE antibodies (representative data of selected antibodies are shown)

To build upon the clinical benefits of anti-HER2 antibodies and reports of anti-tumor activities of IgEs recognising melanoma and ovarian cancer antigens [18, 19, 24], we sought to generate and evaluate a panel of HER2-targeting IgE antibodies. We carried out a stepwise phage display strategy, starting with a screening to identify binders to human HER2 (hHER2), while excluding recognition of human HER1, and followed by selection of binders to rat HER2 (rHER2). This generated 1840 scFv clones, of which 136 were confirmed by ELISA to bind hHER2 (Fig. 1A, B). The top 30 hHER2 binders were engineered into full-length human IgE antibodies, and reactivity to hHER2 was confirmed by ELISA (Fig. 1C). SDS polyacrylamide gel electrophoresis (SDS-PAGE), under reduced and non-reduced conditions, and size-exclusion high performance liquid chromatography (SEC-HPLC) demonstrated the presence of monodisperse product. Antibodies displayed the expected IgE subunit composition and molecular mass for all IgEs. Purity was typically above 95%, even prior to complete removal of by-products and impurities by SEC polishing (Fig. 1D, E).

**Fig. 2:**
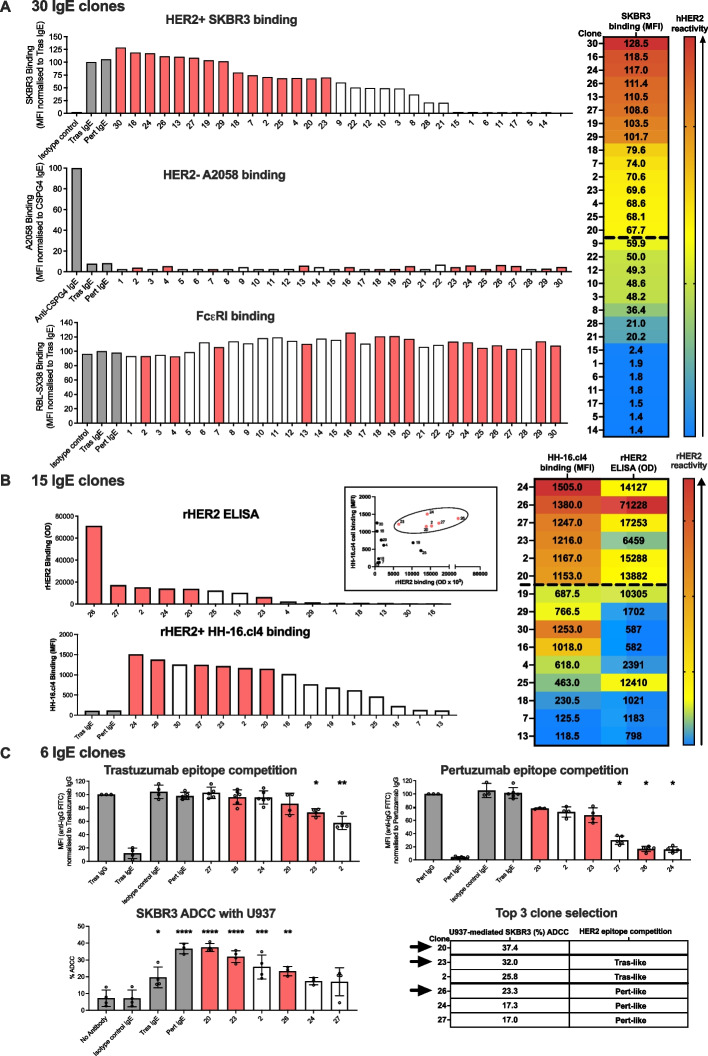
Selection of antibodies based on human HER2 specificity, rat HER2 cross-reactivity and preliminary effector function. **A** (Left). Assessment of binding to hHER2-expressing SKBR3 breast cancer cells (top, *n* = 3–4), lack of binding to hHER2- hCSPG4 + A2058 melanoma cells (middle, *n* = 2); data are presented as MFI normalised to Trastuzumab IgE or CSPG4 IgE positive controls, respectively. Fc binding to human FcεRI-expressing RBL-SX38 cells (bottom). Based on hHER2 specificity 15 antibodies were selected (heatmaps, right), and data for these antibodies is presented in red (bar graphs, left). **B**. The 15 IgEs were assessed for rHER2 cross-reactivity by ELISA (top, representative data), and by flow cytometry to measure binding to rHER2-expressing HH-16.cl4 cells (bottom, *n* = 2, data presented as MFI without normalization). The top six IgE antibodies (presented in red (left)) that showed rHER2 cross-reactivity by ELISA and flow cytometry (inset graph (left)), and heatmap (right) were selected for subsequent functional analyses. **C**. Competition assays showed restriction of Trastuzumab binding to SKBR3 cells by IgEs 2 and 23 indicating their binding epitopes in HER2 domain IV (Trastuzumab-like antibodies) (upper left), and restriction of Pertuzumab binding by IgEs 24, 26 and 27 indicating binding epitopes in HER2 domain II (Pertuzumab-like antibodies) (upper right). Compared to cells alone and isotype control IgE, anti-HER2 IgE antibodies mediated significant ADCC of HER2-expressing SKBR3 (Herceptest 3 +) cells by human U937 monocytic cells (lower left; *n* = 4). Based on HER2 epitope domain determination (top) and human Fc-mediated effector functions (bottom left), three candidate IgE antibodies (table, bottom right) with different epitope recognition characteristics were selected. Selected IgE antibodies are presented in red. Data shown as mean ± SD. Source data are provided as a Source Data file. One-way ANOVA (C) *p ≤ 0.05; **p ≤ 0.01; ***p ≤ 0.001; ****p ≤ 0.0001

We next evaluated recognition of hHER2 antigen in its native form expressed on the cancer cell surface by the IgE antibodies. Flow cytometry screening showed a range of binding abilities to hHER2-expressing SKBR3 breast cancer cells (Herceptest 3 +). None of the antibodies bound to hHER2-negative A2058 melanoma cells, while an IgE specific for the melanoma antigen chondroitin sulfate proteoglycan 4 (CSPG4) was shown to bind to these CSPG4-expressing cells. Evaluation of Fc region binding properties to immune cell surface FcεR by flow cytometry confirmed that all 30 anti-HER2 IgEs bound human FcεRI-expressing rat basophilic leukemia cells (RBL-SX38). Based on hHER2 binding, we selected 15 IgE antibodies for further study (Fig. 2A).

Since the human and rat immune systems demonstrate comparable FcεR expression and cellular distribution, the efficacy and safety of IgE antibodies, including of the first-in-class IgE, MOv18, have been studied in fully immunocompetent syngeneic rat models of cancer [20, 24, 32]. To allow for the possibility to test anti-HER2 IgE in a corresponding rat breast cancer model, the sequential phage display panning approach was adopted to enrich for antibodies exhibiting cross-reactivity to rHER2 (Fig. 1A). We evaluated binding of the selected 15 IgE antibodies to rHER2 by ELISA and to cell surface rHER2 on rat HH-16.cl4 mammary breast cancer cells. These identified six anti-HER2 IgEs that demonstrated cross-reactivity to rHER2 for further in vitro epitope binding domain determination and functional characterisation (Fig. 2B).

To obtain an early indication of HER2 domain recognition, each IgE was subjected to epitope competition assays, performed by flow cytometry in the presence of Trastuzumab and Pertuzumab (in IgG format) (Supplementary Fig. 1). Trastuzumab binds to domain IV of HER2, and Pertuzumab recognizes domain II [33]. IgEs 2 and 23 restricted binding of Trastuzumab IgG, indicating that their binding epitopes are in the area of HER2 domain IV (Trastuzumab-like antibodies). IgEs 24, 26 and 27 restricted binding of Pertuzumab, indicating that their binding epitopes reside in the HER2 domain II area (Pertuzumab-like antibodies). IgE 20 did not significantly restrict either Trastuzumab or Pertuzumab binding, suggesting that the epitope site may reside away from these HER2 sites (non-Trastuzumab or Pertuzumab-like binder) (Fig. 2C, top). Since HER2 auto-phosphorylation is required for activation of pro-tumor processes such as proliferation and migration [34], we evaluated pHER2 inhibitory capacity of the anti-HER2 IgE antibodies. Pertuzumab-like IgEs 24, 26 and 27 significantly reduced HER2 phosphorylation in the presence of the ligand epidermal growth factor (EGF) (Supplementary Fig. 2).

To gain early insight into Fc-mediated effector functions against tumor cells as a key antibody mechanism, [10, 19, 20, 35] we investigated the in vitro effector functions of the six selected anti-HER2 IgE antibodies. IgEs 2, 20, 23 and 26 demonstrated significant induction of ADCC against HER2 + SKBR3 cells, in contrast to isotype control (Fig. IgEs 2, 20, 23 and 26 demonstrated significant induction of antibody-dependent cellular cytotoxicity (ADCC) against HER2 + SKBR3 cells, in contrast to isotype control (Fig. 2C, bottom).

We selected three anti-HER2 antibody candidates representing different epitope reactivities, one Trastuzumab-like (IgE 23), one Pertuzumab-like (IgE 26) and one that binds to neither site (IgE 20), and according to the ranking of in vitro effector functions.

### In vitro Fc-mediated immune effector functions and early assessment of basophil activation of selected IgE antibodies

**Fig. 3:**
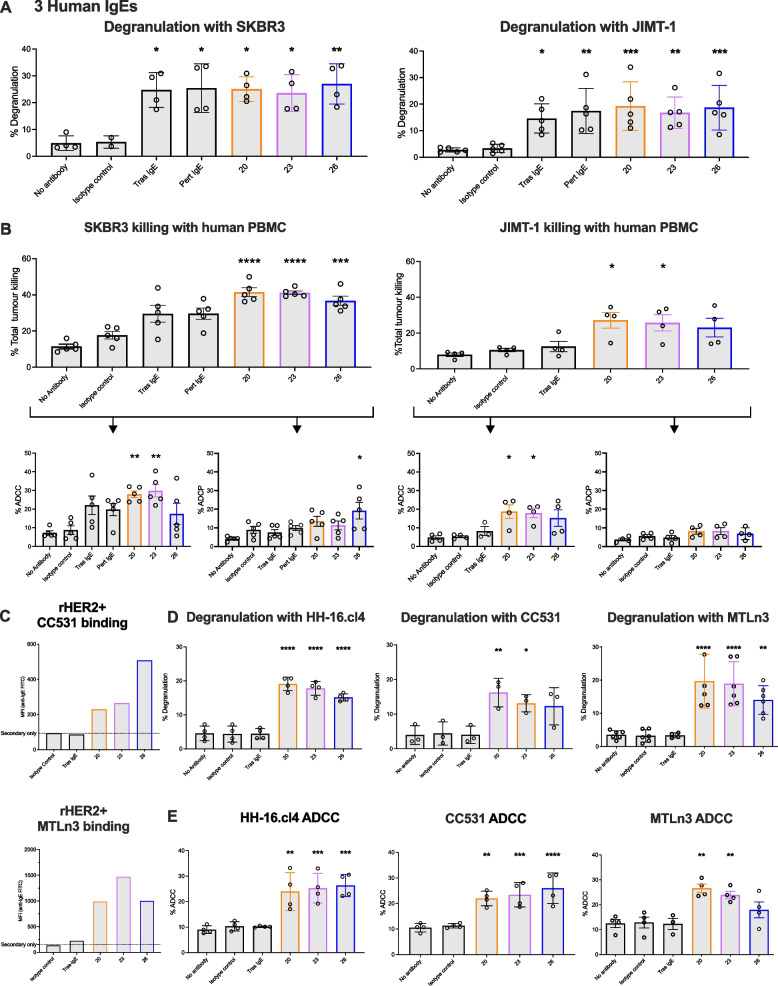
Evaluation of anti-HER2 IgE antibody Fc-mediated effector functions and of basophil activation. **A**. Degranulation of FcεRI-expressing RBL-SX38 cells mediated by all three selected anti-HER2 IgE antibodies cross-linked by HER2-expressing SKBR3 and JIMT-1 breast cancer cells (*n* = 4 and *n* = 5, respectively). **B**. Compared to cells alone and isotype control IgE, anti-HER2 human IgE antibodies mediated significant tumor cell killing (total killing, top; ADCC or ADCP, bottom) of hHER2-expressing SKBR3 (Herceptest 3 +) and JIMT-1 (Herceptest 2 +) cells by human PBMCs (both *n* = 5). **C**. All three anti-HER2 human IgEs bound rHER2-expressing CC531 (top) and MTLn3 (bottom) rat cancer cells (representative flow cytometric binding MFI data shown). **D**. Degranulation of FcεRI-expressing RBL-SX38 cells was mediated by human IgEs when cross-linked by rHER2-expressing HH-16.cl4 (left, *n* = 4), CC531 (middle, *n* = 3), and MTLn3 (right, *n* = 4–6) rat cancer cells. **E**. The human IgEs mediated significant ADCC of HH-16.cl4 (left, *n* = 4), CC531 (middle, *n* = 4) cells by RBL-SX38 and of MTLn3 cells by PBMCs (right, *n* = 4). Data shown as mean ± SD or SEM. Source data are provided as a Source Data file. One-way ANOVA (**A, B, D **and** E**) *p ≤ 0.05; **p ≤ 0.01; ***p ≤ 0.001; ****p ≤ 0.0001

We evaluated the potential of the three selected IgEs to induce Fc-mediated effector functions in the presence of human cancer cells expressing high and medium/low cell surface levels of HER2. We demonstrated comparable binding of the three anti-HER2 human IgEs to human FcεRI and hHER2 (Supplementary Fig. 3). When bound via their Fc domains to human FcεRI-expressing rat basophilic RBL-SX38 cells, all three IgE antibodies triggered significant cell degranulation (measured by β-hexosaminidase release) in the presence of hHER2-expressing SKBR3 (Herceptest 3 +) and JIMT-1 (Herceptest 2 +) cancer cells. As expected, the non-specific isotype control (hapten-specific NIP IgE) did not induce degranulation (Fig. 3A). When exposed to SKBR3 cancer cells, all three IgE antibodies exhibited significant tumor cell killing in the presence of human peripheral blood mononuclear cells (PBMCs), as compared to isotype control. Human IgEs 20 and 23 exhibited significant antibody-dependent cellular cytotoxicity (ADCC), whereas human IgE 26 showed significant antibody-dependent cellular phagocytosis (ADCP). Similarly, the IgEs mediated ADCC of JIMT-1 cells, although statistical significance, as compared to isotype control, was not achieved for IgE 26 (Fig. 3B).

Together, these findings demonstrated the HER2-specific Fc-mediated functions of the three selected IgE antibodies.

Mouse models present immunological limitations for IgE immunotherapy, such as the lack of cross-reactivity of human IgE and murine FcεRs. In contrast, FcεR expression and distribution in rat immune cells is comparable to humans, rendering rats as suitable surrogates for the study of human IgE biology [24–26]. We have shown that the human anti-HER2 IgE antibodies cross-react with rHER2 (Fig. 2B). Therefore, we investigated the anti-tumoral effector functions of the three anti-HER2 IgEs against rHER2-expressing cell lines. In addition to HH-16 cl.4 cells (Fig. 2B), all three human IgEs showed binding to both rat CC531 colon adenocarcinoma and rat MTLn3 mammary adenocarcinoma cells (Fig. 3C). In contrast, we detected no binding of non-specific isotype control IgE, or Tras IgE which does not cross-react with rHER2 [36]. In comparison to isotype control IgE, the human IgE antibodies triggered significant RBL-SX38 cell degranulation in the presence of all three rHER2-expressing rat cancer cells (Fig. 3D) and mediated significant ADCC against these rat cancer cells by either RBL-SX38 cells or PBMCs (Fig. 3E, Supplementary Fig. 4A). No phagocytosis was mediated by any of the antibodies (Supplementary Fig. 4B).

These data suggested that the three selected anti-HER2 IgE antibodies can exert anti-tumor functions against rat cancer cells in vitro.

### Rat IgE antibody functions in vitro and in vivo efficacy in a surrogate rat model of HER2 + breast cancer

**Fig. 4:**
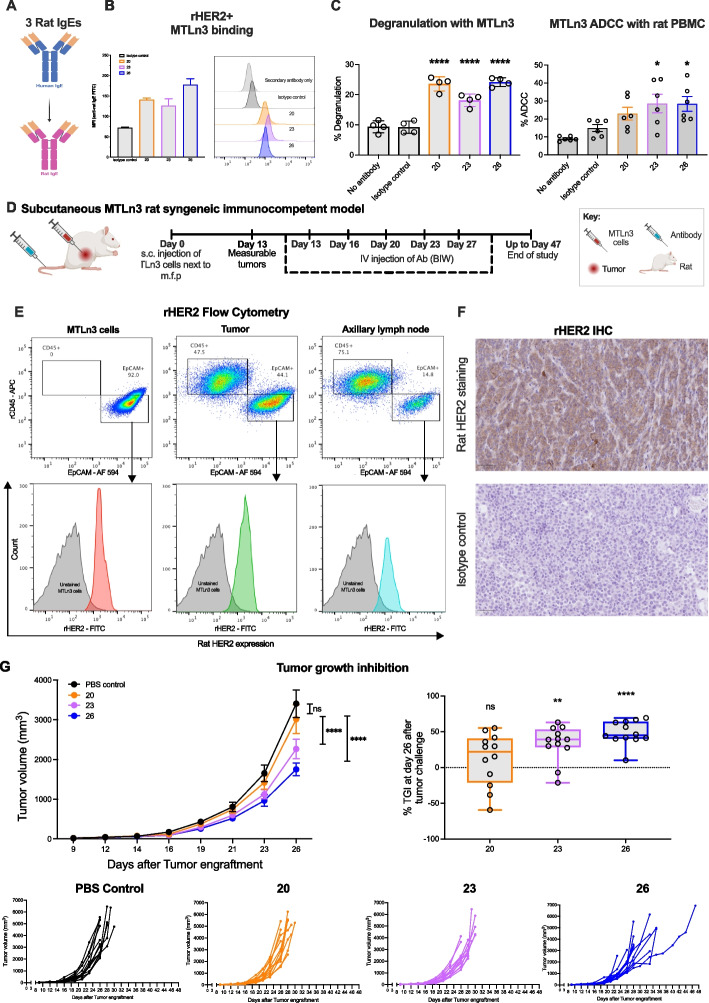
Evaluation of anti-HER2 rat IgEs in vitro and in rat breast cancer in vivo model. **A**. The three selected anti-HER2 antibodies were engineered with rat IgE Fc domains. **B**. Binding of rat IgE antibodies to rHER2-expressing MTLn3 cells was confirmed by flow cytometry. **C**. All three rat IgEs triggered degranulation of FcεRI-expressing RBL-SX38 cells when cross-linked by MTLn3 cells (left, *n* = 4), and rat IgEs 23 and 26 mediated ADCC of MTLn3 cells by rat PBMCs (right, *n* = 6). **D**. In vivo study design and anti-HER2 IgE dosing schedule. **E**. Flow cytometric analyses (top: dot plots; bottom: histograms) of rHER2 expression on EpCAM + MTLn3 cells from in vitro cell cultures (left), and on EpCAM + tumor cells isolated from MTLn3 tumor and enlarged axillary lymph node (middle and right, respectively), harvested at the end of study. **F**. Immunohistochemical staining of MTLn3 tumors (excised at the end of study) with rHER2 antibody (top) and isotype control antibody (bottom) (representative images shown; 10 × magnification; scale bar = 50 μm). **G**. Anti-HER2 rat IgEs 23 and 26 significantly inhibited MTLn3 tumor growth. Rats were treated with PBS control (black, *n* = 14), or rat IgEs 20 (orange, *n* = 12), 23 (lilac, *n* = 13), or 26 (blue, *n* = 13). Top left: tumor volume measurements over time. Top right: tumor growth inhibition mediated by the rat IgE antibodies. Bottom panel: graphs showing tumor growth curves for individual animals. Data are shown as mean ± SD (**C**, left) or SEM (**C**, right and **G**). Source data are provided as a Source Data file. One-way ANOVA (**C** and **G**, right), Two-way ANOVA (**G**, left; day 26 analyses shown; full statistical analyses shown in Supplementary Table 1) *p ≤ 0.05; **p ≤ 0.01; ***p ≤ 0.001; ****p ≤ 0.0001

In order to study the functions of the IgE antibodies in a surrogate rat model of cancer, we generated the three IgEs with rat IgE Fc domains (rat IgEs 20, 23, 26) (Fig. 4A). We confirmed affinity of the three anti-HER2 rat IgE antibodies to human FcεRI and hHER2 (Supplementary Fig. 5) and binding to rHER2-expressing MTLn3 breast cancer cells (Fig. 4B). We furthermore demonstrated Fc-mediated effector functions against rat MTLn3 breast cancer cells in vitro. All three rat IgE antibodies triggered significant degranulation of RBL-SX38 cells in the presence of MTLn3, compared to the IgE isotype control (Fig. 4C, left). Rat IgEs 23 and 26 mediated significant ADCC of MTLn3 cells by rat PBMCs, above isotype control IgE. No ADCP was mediated by any of the IgEs (Fig. 4C right, Supplementary Fig. 4C).

We next evaluated the potential of IgE antibodies to restrict in vivo tumor growth in a syngeneic rHER2-expressing immunocompetent model of breast cancer. Fischer rats were challenged subcutaneously (*s.c.*) with syngeneic rat MTLn3 cells and then treated with rat IgEs bi-weekly (BIW) (Fig. 4D). Tumors excised at the end of the study retained rHER2 expression comparable to in vitro cultured MTLn3 cells, as confirmed by flow cytometric analysis (Fig. 4E). rHER2 expression of developed tumors was also confirmed by immunohistochemistry (Fig. 4F). While tumor growth in rats treated with rat IgE 20 was comparable to untreated (PBS control) animals, rat IgEs 23 and 26 mediated significant tumor growth inhibition (TGI) (Fig. 4G, top panel). The individual tumor growth curves for each animal in different treatment groups are shown in Fig. 4G (bottom panel). Potential adverse events (AEs) in this immunocompetent model were monitored after each antibody dose. Mild AEs were observed across the three anti-HER2 IgE treated groups. These were transient, resolving within 1 h, and with no overall impact on rat body weights (Supplementary Fig. 6).

Together, these findings demonstrate in vitro Fc-mediated effector functions of the anti-HER2 rat IgE antibodies against rHER2 + MTLn3 rat mammary adenocarcinoma cells. In vivo*,* anti-HER2 rat IgE antibodies were well tolerated in immunocompetent hosts, and rat IgEs 23 and 26 mediated significant growth restriction of MTLn3 tumors.

### Intra-tumoral immune cell infiltration in MTLn3 tumors following treatment with anti-HER2 IgE

**Fig. 5:**
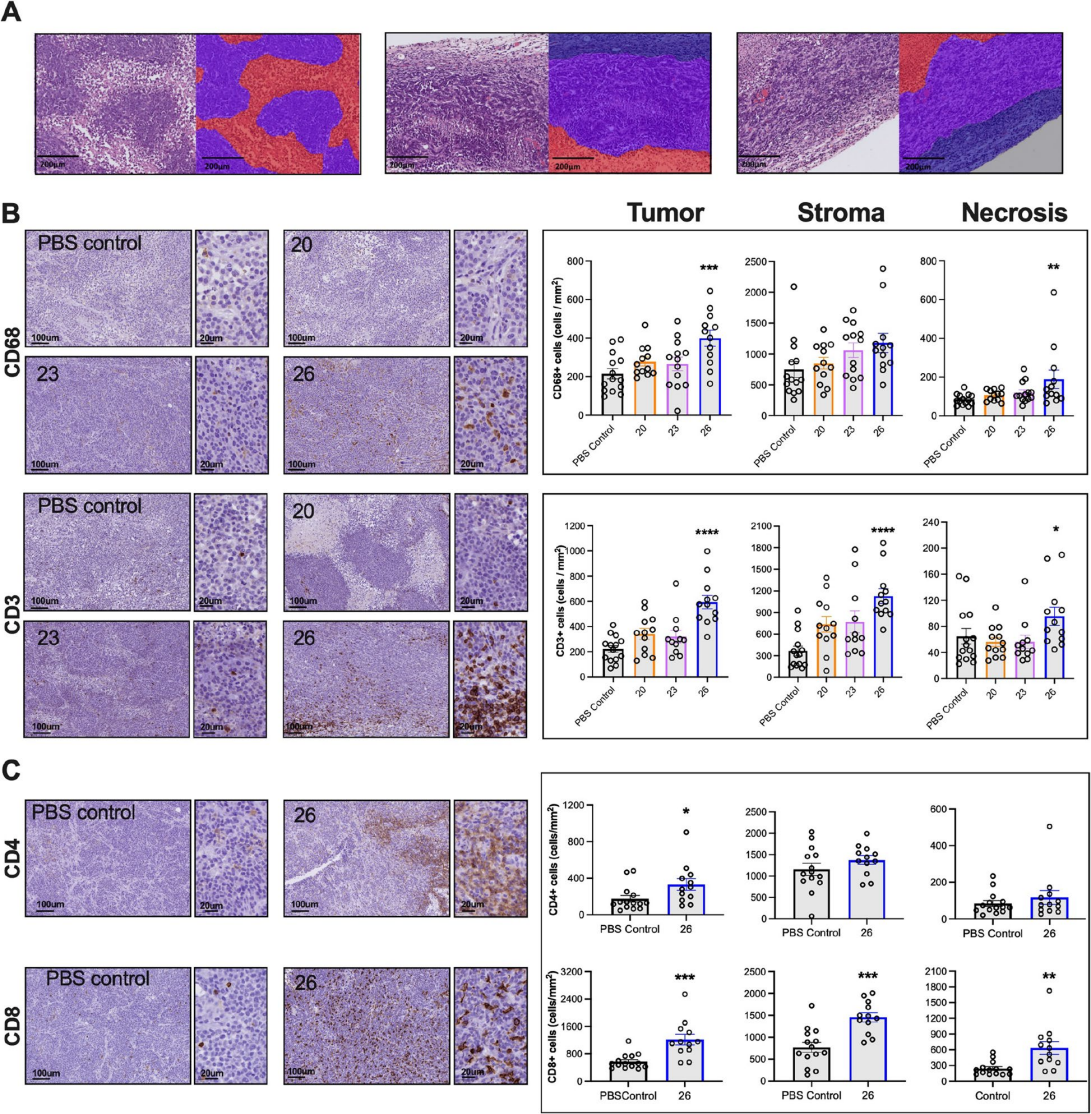
Immune cell infiltration into breast tumors from rats treated with anti-HER2 IgE. **A.** Representative images of MTLn3 tumor tissues from end of study. Demarcated areas of tumor (purple), stroma (blue) and necrosis (red) prior to evaluation of immune cell infiltration using image analysis. 10 × magnification; scale bars = 200 μm **B**. Left panels: representative images of CD68 + and CD3 + stained tumors of animals treated with PBS, rat 20, 23 and 26 IgE. Right graphs: immunohistochemical analyses show significantly increased infiltration of CD68 + and CD3 + immune cells in rat 26 IgE-treated animals, compared to PBS group. **C.** Left panels show representative images of CD4 + and CD8 + stained tumors of animals treated with PBS or rat 26 IgE, while the right graphs present the corresponding immunohistochemical analyses. IgE treatment was associated with increased infiltration of CD4 + and CD8 + cells compared with tumors from PBS controls. **B** and **C** left: 10 × magnification with 40 × magnified inset; scale bars = 100 μm and 20 μm, respectively. **B** and **C** Right: Bar graphs showing quantification of cell infiltration (presented as cell density, cells/mm^2^; PBS, *n* = 14, rat IgEs 20, *n* = 12; 23, *n* = 13 and 26, *n* = 12). One section per tumor/animal for each marker was analysed. Data are shown as mean ± SEM. Source data are provided as a Source Data file. Unpaired t-test, Kruskal–Wallis or Mann–Whitney tests (**B** and** C**, right) *p ≤ 0.05; **p ≤ 0.01; ***p ≤ 0.001; ****p ≤ 0.0001

To evaluate immune cell infiltration in the rHER2-expressing tumors following IgE treatment, we performed immunohistochemical analyses of tumoral, stromal and necrotic areas (Fig. 5A). Tumors from rat IgE 26-treated animals showed significantly increased infiltration of CD68^+^ macrophages in tumor and necrotic areas and of CD3^+^ T cells in tumoral, stromal and necrotic areas, compared to tumors from PBS controls (Fig. 5B).

Having shown superior in vivo efficacy and intra-tumoral immune cell infiltration following treatment with rat IgE 26, we carried out additional analyses of tumors treated with this antibody. These revealed that in addition to CD3 + T cells, IgE treatment was associated with significantly increased CD8^+^ T cells in tumor, stromal, and necrotic areas, while CD4^+^ T cell infiltration was significantly increased in tumor areas, as compared to PBS controls (Fig. 5C).

**Fig. 6:**
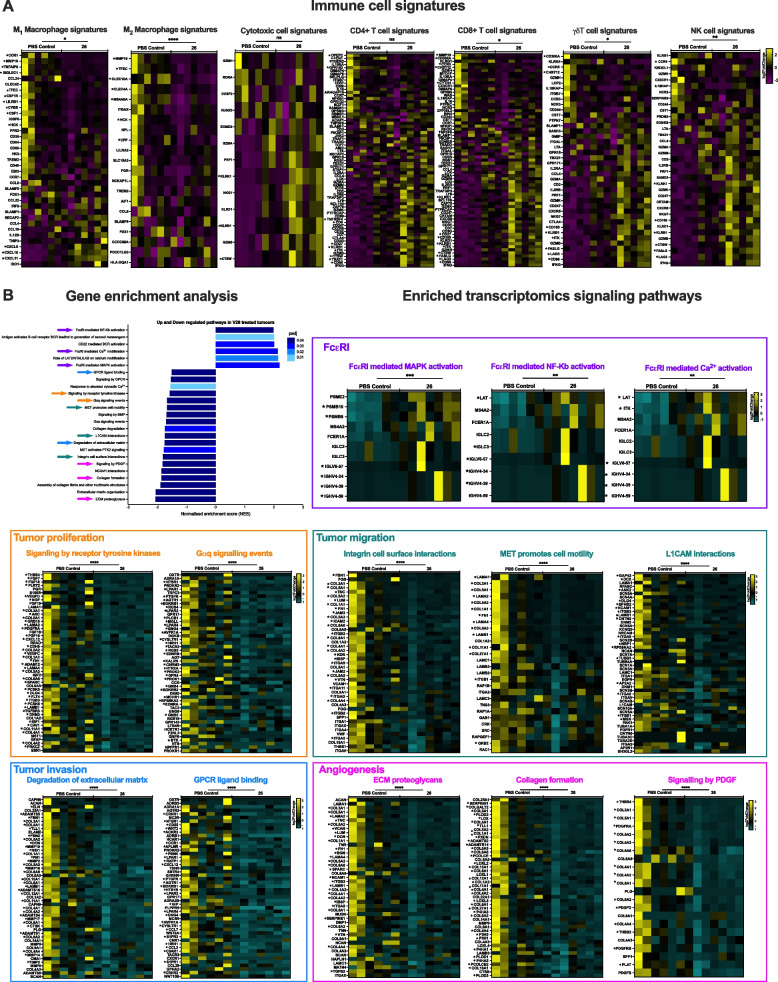
Transcriptomic profile of anti-HER2 IgE-treated rat tumors: immune cell signatures and pathways analysis. Gene expression and pathway analyses were studied in MTLn3 tumors excised from rats treated with anti-HER2 rat IgE 26 (*n* = 7) or PBS controls (*n* = 6). Upregulated and downregulated genes are presented in yellow and magenta, respectively. **A**. Significantly enriched expression, evaluated according to ConsensusTME database (https://github.com/cansysbio/ConsensusTME/tree/master/Consensus_Signatures) for breast cancer, of M2 macrophage, CD8 + T cell, γδT cell and NK cell gene signatures, were observed in tumors from animals treated with rat IgE 26. **B**. Gene set enrichment analysis using the package fgsea, identified differential expressed genes ranked according to fold change and calculated enrichment of gene sets was evaluated within Reactome. Selected examples of pathways are denoted with arrows, including: FcεRI (purple), tumor invasion (blue), tumor proliferation (orange), tumor migration (teal), tumor angiogenesis (pink). Differentially expressed (FDR corrected) genes are shown for each selected pathway (FcɛRI; *n* = 9, *n* = 10, *n* = 11; tumor proliferation: *n* = 38, *n* = 23; tumor migration *n* = 30, *n* = 13, *n* = 18, tumor invasion *n* = 33, *n* = 28; angiogenesis *n* = 28, *n* = 37, *n* = 17). Upregulated and downregulated genes are presented in yellow and teal, respectively. Full statistical analyses were performed using False discovery rate (FDR) test and are shown in Supplementary Tables 2 and 3 for each gene. *p ≤ 0.05. Data are shown as log2FoldChange. Source data are provided as a Source Data file. Wilcoxon test was applied to each gene signature list to compare rat IgE 26 and PBS treated animals (**A** and **B** heatmaps); *p ≤ 0.05; **p ≤ 0.01; ***p ≤ 0.001; ****p ≤ 0.0001

Transcriptomic analysis of tumors excised from rat IgE 26-treated animals revealed downregulation of M2 macrophage-associated genes (Fig. 6A). This suggested a switch away from immunoregulatory phenotypes in the TME. In agreement with our immunohistochemical analyses (Fig. 5), upregulation of gene signatures for cytotoxic, CD4^+^, CD8^+^, γδT and NK cells were also found in tumors from rat IgE 26-treated animals compared to controls (Fig. 6A). These data were also supported by deconvolution analysis which indicated higher abundance of these cell types in tumors from treated rats, as compared with controls (Supplementary Fig. 7). Gene set enrichment analysis revealed that treatment with rat IgE 26 was associated with upregulation of FcɛRI signalling pathways and downregulation of numerous pathways associated with tumor progression, including proliferation, invasion, migration, and angiogenesis (Fig. 6B). Additionally, we explored whether rat IgE 26 also displayed Fab-mediated direct effects, which may contribute to the observed in vivo tumor growth restriction. In vitro assays showed that rat IgE 26 significantly inhibited tumor cell colony formation and migration of MTLn3 cells, compared to isotype control IgE (Supplementary Fig. 8).

**Fig. 7:**
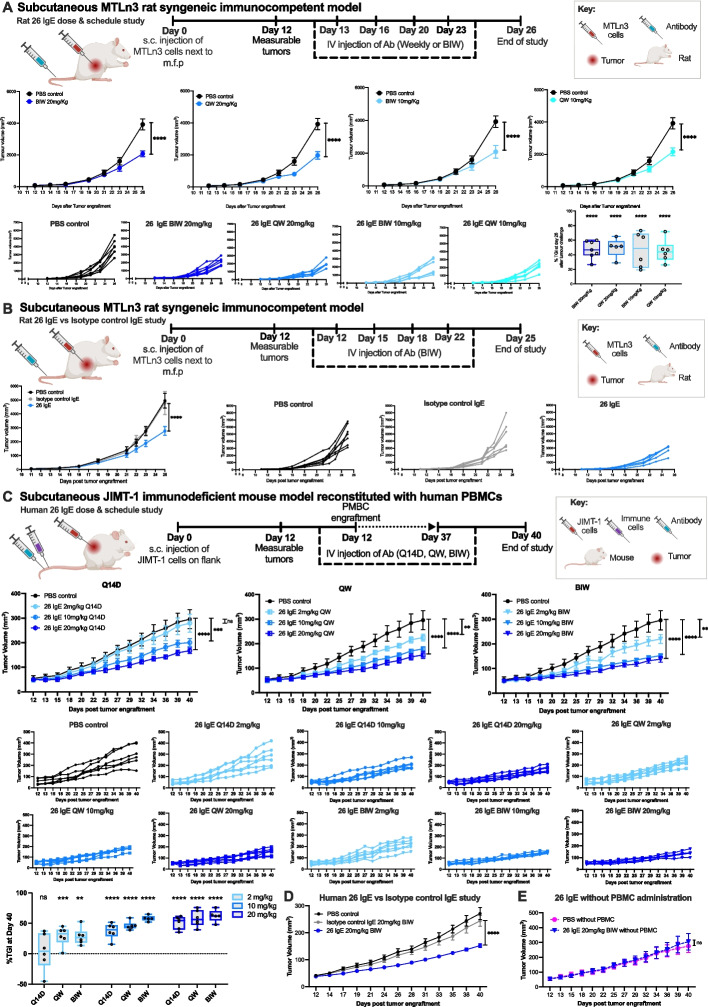
Further in vivo evaluations of anti-HER2 IgE in rat and human xenograft breast cancer models. **A**. Top panel: Rat in vivo dose and scheduling study design. Middle panel: Rat IgE 26 significantly inhibited the growth of MTLn3 tumors at both 10 and 20 mg/kg doses administered either weekly or bi-weekly (cyan, *n* = 6; light blue, *n* = 6; medium blue, *n* = 6; dark blue, *n* = 7; respectively) compared to PBS controls (black, *n* = 7). Bottom panels: Individual tumor growth curves and tumor growth inhibition (right). **B**. Top panel: Study design for IgE 26 and IgE isotype control antibody treatments. Bottom panel: Rat IgE 26 (blue, *n* = 5) significantly inhibited MTLn3 in vivo growth compared to non-specific isotype control IgE (grey, *n* = 6) (left); tumor growth curves for individual animals (right; PBS, black, *n* = 7). **C**. Top panel: JIMT-1 human breast cancer xenograft model study design. Middle panels: Human IgE 26 significantly inhibited the growth of JIMT-1 tumors in human PBMC-engrafted mice at 10 and 20 mg/kg doses administered at all dosing frequencies, and at 2 mg/kg dose administered weekly and bi-weekly, as compared to PBS control (black) (all groups *n* = 6). Each lower graph shows tumor growth curves for individual animals. Bottom left panel: Tumor growth inhibition for each dose and schedule regimen. **D**. Human IgE 26 administered bi-weekly at 20 mg/kg (blue) significantly inhibited JIMT-1 in vivo growth compared to non-specific isotype control IgE at the same dose and frequency (grey) (all groups *n* = 12). **E.** Human IgE 26 (blue) did not significantly restrict JIMT-1 tumor growth in mice lacking human PBMC engraftment, compared to PBS controls (pink) (all groups *n* = 6). Data shown as mean ± SEM. Two-way ANOVA (**A**, top; **B**, left; **C**, top and bottom left; **D**; and **E**; analyses shown for the day of end of study; full statistical analyses shown in Supplementary Tables 4—11), One way-ANOVA (**A**, bottom right) *p ≤ 0.05; **p ≤ 0.01; ***p ≤ 0.001; ****p ≤ 0.0001

To consider the translation of IgE 26, we performed dosing and isotype control comparative studies. Treatment with rat IgE 26 at 10 mg/kg and 20 mg/kg, administered either weekly or bi-weekly, showed significant TGI when compared to untreated control rats (Fig. 7A). Target-specific anti-tumor effects of rat IgE 26 (7 mg/kg bi-weekly) were confirmed by significantly inhibited tumor growth, as compared to non-specific IgE isotype control and PBS treatments (Fig. 7B).

Together, these findings suggest that different systemic dosing regimens of rat IgE 26 mediated significant tumor restriction in an immunocompetent syngeneic rat model of breast cancer. Treatment was associated with recruitment of pro-inflammatory immune cells into the TME, along with increased FcɛRI signalling and cytotoxic immune cell signatures. This was accompanied by suppression of pro-tumoral pathways involved in proliferation, invasion, migration, and angiogenesis.

### In vivo efficacy of human anti-HER2 IgE in a human breast cancer xenograft model resistant to Fab-mediated antibody effects

To extend our investigation of the efficacy of the human version of the lead anti-HER2 IgE 26, we sought to explore anti-tumor activity in a human breast cancer xenograft model, known to be resistant to the Fab-mediated effects of Trastuzumab [8, 9, 33]. First, we confirmed resistance of JIMT-1 human breast cancer cells to Fab-mediated functions of human IgE 26 in vitro (Supplementary Fig. 9). Immunodeficient mice were engrafted with PBMCs and challenged subcutaneously with hHER2-expressing JIMT-1 breast cancer cells. Mice were then treated intravenously with human IgE 26 at 2, 10 or 20 mg/kg, either fortnightly (Q14D), weekly (QW) or bi-weekly (BIW). Tumor growth restriction was significantly greater in mice treated with 10 mg/kg and 20 mg/kg doses when compared to PBS controls, regardless of treatment frequency. Furthermore, at 2 mg/kg, significant efficacy was observed with QW and BIW dosing (Fig. 7C). Target-specific efficacy of human IgE 26 (20 mg/kg BIW) was confirmed by significant tumor growth restriction, as compared to non-specific IgE isotype control and PBS treatments (Fig. 7D). Furthermore, the anti-tumor effects of human IgE 26 were observed only when treatment was accompanied by PBMC effector cell administration (Fig. 7E).

This data confirms immune-mediated functions as the key contributor to the anti-tumoral activity of IgE immunotherapy.

## Discussion

HER2-expressing breast cancer is known for its aggressive nature and poor prognosis. While IgG antibody-based therapies such as Trastuzumab and Pertuzumab initially demonstrate promising clinical outcomes, the emergence of various resistance mechanisms often limits their long-term efficacy and patient benefit [8, 9]. Recent studies have highlighted the potential advantage of harnessing the immune-stimulating properties of IgE in targeting tumor antigens, with a first-in-class IgE showing encouraging signs of clinical efficacy [18]. However, a focused study aimed at selecting an IgE antibody with optimal characteristics amenable for breast cancer treatment has not been conducted.

Here, we demonstrate a novel IgE class antibody therapeutic discovery platform that allows the selection of an immune-stimulating lead candidate targeting HER2, and which could have application across diverse tumor targets. We characterise the anti-tumor functions of three IgE antibodies, selected from the screening of 1840 antibody variable region domains generated against HER2. Our candidate selection of IgEs 20, 23 and 26 was based on biophysical attributes, recognition of human and rat homologues of HER2 (to allow for the development of suitable human and rat breast cancer surrogate models), epitope reactivity, and Fc-mediated effector potency. Trastuzumab binds to domain IV of HER2, and Pertuzumab recognizes domain II [33, 37]. Thus, we selected three IgE antibodies to broadly represent epitope reactivity to HER2 domain IV (Trastuzumab-like IgE 23), and HER2 domain II (Pertuzumab-like IgE 26). We selected IgE 20 because it did not recognise either epitope site. Despite these observations, the cross-reactivity of our novel antibodies with rHER2 suggests distinct binding epitopes from Trastuzumab and Pertuzumab, which are not rat cross-reactive [36]. When we investigated pHER2 inhibitory potential of the anti-HER2 IgE antibodies, IgE 26 engendered significantly reduced ligand-dependent HER2 phosphorylation, corroborant with known functions of Pertuzumab [33, 37].

Immune effector functions are one of the hallmarks of antibody-based therapies. All three IgE antibodies induced degranulation of RBL-SX38 in the presence of hHER2-expressing SKBR3 (Herceptest 3 +) and JIMT-1 (Herceptest 2 +) breast cancer cells. All three IgEs mediated ADCC of these tumour cells by human immune effector cells. These Fc-mediated functions align with findings generated with other tumor-antigen specific IgE antibodies, including those targeting FRα and CSPG4 [11, 19, 20].

A key challenge in designing immunologically relevant mouse models to investigate IgE therapeutic antibody candidates arises from the inability of human IgE to cross-react with murine FcεRI, and from the lack of trimeric FcεRI expression by mouse immune cells such as monocytes, macrophages and others. This renders mouse models unsuitable for recapitulating IgE immunity and thus for evaluating the anti-tumor functions of IgE antibodies in vivo. The rat IgE immune response however, featuring FcεR expression across the same immune cell populations as those found in humans, may better correspond to the human patient setting [25, 26, 38]. Therefore, rat models of syngeneic cancer permit the study of IgE immune-mediated mechanisms against tumors in a fully immunocompetent host. Cross-reactivity of the candidate IgE between human tumor-associated antigen and that of different animal species is an important criterion for selection of antibodies for pre-clinical testing and translation to the clinic [32]. To study anti-HER2 IgE in an appropriate surrogate cancer model, antibody cross-reactivity to the rat homologue of HER2 was an important consideration. Therefore, we verified binding of the IgE antibodies to human and rat HER2-expressing cancer cell lines during the selection of our three chosen candidates for further study. Following engineering of the selected antibodies in the rat Fc format, we confirmed their Fc-mediated functions to engender rat immune cell-mediated killing of rHER2 + breast cancer cells.

Concordantly, evaluation of IgE antibody efficacy in a syngeneic rat model of breast cancer provided evaluations of in vivo potency, as well as insights into the immune-stimulatory functions of IgE in this immunocompetent system. Rat IgE 26 engendered the greatest cancer growth restriction of the three IgE candidates and restricted tumor growth at lower doses and treatment frequencies. The antigen-specific effects were confirmed by comparison with an isotype control IgE. Moreover, in this model, repeated dosing of anti-HER2 rat IgEs to immunocompetent rats did not trigger severe toxicity or overt signs of anaphylaxis, in line with observations for IgE antibodies that target other tumor-associated antigens [19, 20, 35].

Alongside tumor growth restricting effects in the rat model of breast cancer, rat IgE 26 treatment was associated with significant recruitment of CD68 + macrophages into the tumor. Additionally, for the first time, a significant CD3 + T cell infiltration, including of CD4 + and CD8 + T cells, was observed in tumors treated with an antigen-targeting IgE, compared to control animals. These observations were corroborated by transcriptomic analyses showing enrichment of immune cell gene signatures for CD8 + and γδT cells. Moreover, enhanced NK cell signatures and reduced alternatively-activated macrophage signatures were observed in tumors from rat IgE 26-treated animals. Pathway analysis also revealed activation of several immune signalling pathways associated with FcεRI. These point to a significant immunological shift towards a pro-inflammatory environment in the HER2-expressing tumors after IgE treatment. These observations are consistent with previously reported pro-inflammatory re-polarisation of immune responses in the TME following treatment with IgEs recognising different antigens and tumor types [19, 20, 35]. Together, these suggest that IgE immunotherapy may reverse immunosuppressive conditions in tumors. Furthermore, numerous pathways associated with tumor development, including proliferation, migration, invasion, and angiogenesis, were downregulated in tumors from rat IgE 26-treated animals, when compared to controls. These findings, together with in vitro direct effects against breast cancer cells, suggest that IgE 26 may exert a combination of Fab- and Fc-mediated anti-tumoral functions in vivo.

IgG immunotherapies for HER2-expressing tumors can be limited by resistance mechanisms, including alternatively-activated macrophages, which often express inhibitory FcγR2b receptors, restricting IgG anti-tumor effector functions. In contrast, IgE antibodies have no known inhibitory Fc receptor, and can harness these macrophage subsets to exert potent Fc-mediated anti-tumor functions. This may provide a means to overcome known resistance mechanisms [19, 21]. Human IgE 26 also displayed significant efficacy in a humanised mouse model in the presence of human PBMCs. Unlike MTLn3 tumors in the rat immunocompetent model, human JIMT-1 breast cancer cells are resistant to Fab-mediated effects of Trastuzumab [8, 9] and of our human IgE 26. This may suggest that the Fc-mediated immune functions of human IgE 26 may be applied for the treatment of HER2-expressing tumors resistant to trastuzumab Fab-mediated effects. Future work may include studies of the potential mechanisms by which IgE 26 could overcome resistance to anti-tumor therapies.

In different host species and immunological contexts (immunocompetent rat vs immunocompromised mice reconstituted with human immune cells), IgE 26 shows comparable anti-tumor efficacy, and when directed against tumors with medium/low antigen expression. In concordance, another IgE targeting FRα engendered anti-tumor functions targeting low antigen expressing tumors [18, 20].

Together, our findings in both in vivo model systems support the Fc-mediated anti-tumor effects of human IgE, and its corresponding rat IgE, against medium/low HER2-expressing breast cancer.

## Conclusions

In conclusion, we demonstrated that three anti-HER2 IgE antibodies selected from screening of 1840 scFv and 30 full length IgE clones, mediated in vitro anti-tumor activity against breast cancer cells, in both rodent and human immune contexts. IgE 26 showed superior tumor growth restriction in a syngeneic immunocompetent rat model of breast cancer. Efficacy of IgE 26 was recapitulated in a human breast cancer xenograft model in mice. IgE treatment was associated with increased immune cell infiltration, upregulation of several immune-related pathways and downregulation of numerous tumor progression pathways. We unveil a comprehensive platform for IgE antibody lead candidate selection. Our findings highlight IgE engineering technologies as therapeutic opportunities for patient groups with HER2-expressing disease that do not benefit from approved anti-HER2 antibodies.

## Supplementary Information

Supplementary Material 1. Supplementary Fig. 1.pdf – Epitope competition assay: Schematic of the flow cytometric epitope competition assay to evaluate the broad areas on HER2 recognised by each antibody.

Supplementary Material 2. Supplementary Fig. 2.pdf – Evaluation of IgE antibodies to interfere with the phosphorylation of HER2. SKBR3 were incubated in the presence of hEGF for 30 min prior addition of IgE antibodies at 5 µg/ml, resulting in varying levels of inhibition of phosphorylation of hHER2 (*n* = 4). Data presented as pHER2 normalised to the total HER2 measured for each condition. Data shown as mean ± SD. Source data are provided as a Source Data file. One-way ANOVA compared to isotype control; ****p ≤ 0.0001.

Supplementary Material 3. Supplementary Fig. 3.pdf – Measurements of affinity of human IgE antibodies for human HER2 and FcεRI. Surface Plasmon Resonance was conducted to evaluate IgE antibody affinity to human recombinant FcεRIα and human recombinant HER2.

Supplementary Material 4. Supplementary Fig. 4.pdf – Evaluation of human and rat IgE antibody-dependent cellular phagocytosis (ADCP) of rat breast cancer cells. (A) Representative flow cytometric ADCC/ADCP assay plots: R1: total (CFSE stained) FITC + tumor cell population; R2: (CFSE stained) FITC + /APC + phagocytosed cells; R3: (CFSE stained) FITC + /DAPI + dead tumor cells. (B) Human IgEs 20, 23 and 26 (*n* = 4) and (C) rat IgE counterparts (*n* = 9) were evaluated for ability to trigger ADCP of rat HER2-expressing MTLn3 breast cancer cells by rat PMBC, measured by flow cytometry. Data shown as mean ± SD. Source data are provided as a Source Data file. One-way ANOVA showed no significant difference between the level of ADCP with controls or test IgE antibodies (B, C).

Supplementary Material 5. Supplementary Fig. 5.pdf – Affinity measurements of rat IgE antibodies for human FcεRI and HER2. Surface Plasmon Resonance was conducted to evaluate rat IgE antibody affinity to human recombinant FcεRIα and human recombinant HER2.

Supplementary Material 6. Supplementary Fig. 6.pdf – Assessment of toxicity with IgE administration immunocompetent syngeneic rat model of HER2-expressing MTLn3 breast cancer in vivo. Summary of adverse events observed with intravenous administration of MTLn3 tumor bearing rats with either PBS (control), rat IgEs 20, 23 or 26, shown for each treatment condition and across different doses. Bottom right graph: animal weight measurements for each treatment group over time until termination.

Supplementary Material 7. Supplementary Fig. 7.pdf – Transcriptomic analyses of tumor specimens from rats treated with anti-HER2 IgE. Deconvolution analyses revealed differential abundance of M2 macrophages, cytotoxic cells, and NK cells in tumors from animals treated with rat IgE 26. Welch’s t-test; ns not significant; *p ≤ 0.05.

Supplementary Material 8. Supplementary Fig. 8.pdf – Fab-mediated direct effects of rat IgE antibodies against MTLn3 cancer cells in vitro. The rat IgEs 20, 23 and 26 were evaluated for ability to affect different tumor cell mechanisms compared with IgE isotype control treatment in vitro. Antibody effects were measured on colony formation (A) and migration (B). Top: Bar graphs show colony formation and cellular migration measurements from 6 and 4 independent experiments, respectively. Bottom: Representative images of colony formation and migration. Data shown as mean ± SD. Source data are provided as a Source Data file. One-way ANOVA (A, B) *p ≤ 0.05; ***p ≤ 0.001.

Supplementary Material 9. Supplementary Fig. 9.pdf – Confirmation of resistance to Fab-mediated direct effects of JIMT-1 human breast cancer cells. JIMT-1 human breast cancer cells show resistance to Fab-mediated functions of human IgE 26 in a ligand-independent cell viability in vitro assay (*n* = 4). Data shown as mean ± SD. Source data are provided as a Source Data file. Two-way ANOVA showed no significant difference between isotype control and V26 IgE at any concentration.

Supplementary Material 10: Supplementary Table 1. Statistical analysis of rat anti-HER2 IgEs efficacy study in immunocompetent syngeneic rat model of HER2-expressing MTLn3 breast cancer.

Supplementary Material 11: Supplementary Table 2. Significantly differently-expressed genes (DEGs) in transcriptomic analyses of immune signatures within rat IgE 26-treated tumor specimens from immunocompetent syngeneic rat model of HER2-expressing MTLn3 breast cancer.

Supplementary Material 12: Supplementary Table 3. Significantly differently-expressed genes (DEGs) in transcriptomic analyses of signalling pathways within rat IgE 26-treated tumor specimens from immunocompetent syngeneic rat model of HER2-expressing MTLn3 breast cancer.

Supplementary Material 13: Supplementary Table 4. Statistical analysis of rat IgE 26 dose and scheduling study in immunocompetent syngeneic rat model of HER2-expressing MTLn3 breast cancer.

Supplementary Material 14: Supplementary Table 5. Statistical analysis of rat IgE 26 compared to isotype control IgE in immunocompetent syngeneic rat model of HER2-expressing MTLn3 breast cancer.

Supplementary Material 15: Supplementary Table 6. Statistical analysis of human IgE 26 dose and scheduling study in a human breast cancer xenograft model (Q14D).

Supplementary Material 16: Supplementary Table 7. Statistical analysis of human IgE 26 dose and scheduling study in a human breast cancer xenograft model (QW).

Supplementary Material 17: Supplementary Table 8. Statistical analysis of human IgE 26 dose and scheduling study in a human breast cancer xenograft model (BIW).

Supplementary Material 18: Supplementary Table 9. Statistical analysis of human IgE 26 compared to isotype control IgE in a human breast cancer xenograft model.

Supplementary Material 19: Supplementary Table 10. Statistical analysis of human IgE 26 + PBMCs in a human breast cancer xenograft model.

Supplementary Material 20: Supplementary Table 11. Statistical analysis of human IgE 26 -PBMCs in a human breast cancer xenograft model.

## Data Availability

The data that support the findings of this study are available from the corresponding author upon reasonable request. Publicly available datasets used in this study are: The Reactome Pathway Database (https://reactome.org) and Consensus^TME^database,BRCA_consensus_Signatures (https://github.com/cansysbio/ConsensusTME/blob/master/).

## Abbreviations

HER2: Human epidermal growth factor receptor 2
ER: Estrogen receptor
IHC: Immunohistochemistry
ISH: In situ hybridization
IgG: Immunoglobulin G
FcγRs: Fc-gamma receptors
TME: Tumor microenvironment
IgE: Immunoglobulin E
FcεRI: Fc-epsilon receptor I
FRα: Folate receptor alpha
rHER2 +: Rat epidermal growth factor receptor 2 positive
PBMCs: Peripheral blood mononuclear cells
ADCC: Antibody-dependent cell-mediated cytotoxicity
ADCP: Antibody-dependent cell-mediated phagocytosis
Phospho-HER2: Phosphorylation of HER2
Q14D: Fortnightly
QW: Weekly
BIW: Bi-weekly
DAB: 3,3'-Diaminobenzidine
SEC-HPLC: Size-exclusion high performance liquid chromatography
CSPG4: Chondroitin sulfate proteoglycan 4
TGI: Tumor growth inhibition
GESA: Gene Set Enrichment Analysis
