# Supplementary material for: An IgE antibody targeting HER2 identified by clonal selection restricts breast cancer growth via immune-stimulating activities
Source: J Exp Clin Cancer Res. 2025 Feb 12;44:49. doi: 10.1186/s13046-025-03319-5 (PMC11818027; doi:10.1186/s13046-025-03319-5)
Supplement: Supplementary file 1 — Supplementary Material 1. Supplementary Fig. 1.pdf – Epitope competition assay: Schematic of the flow cytometric epitope competition assay to evaluate the broad areas on HER2 recognised by each antibody. [file 13046_2025_3319_MOESM1_ESM.pdf]

Eptiope competition assay set-up

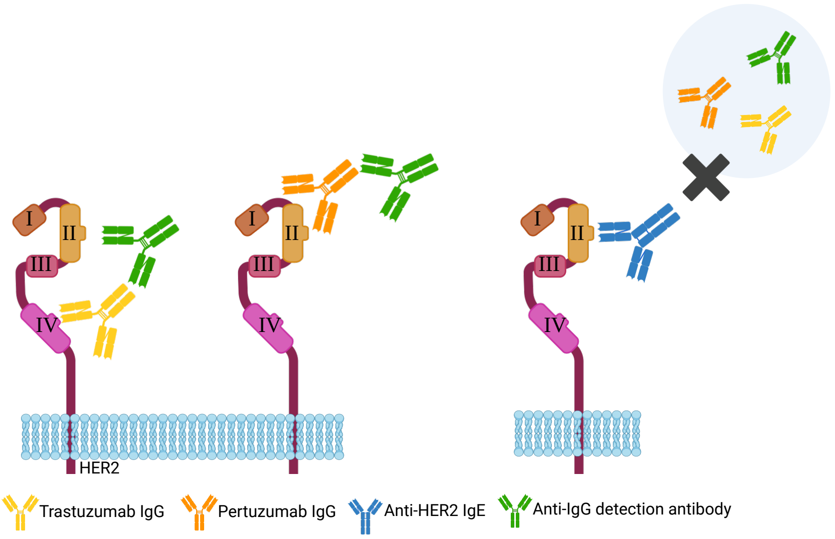

Supplementary Figure 1: Epitope competition assay. Schematic of the flow cytometric epitope competition assay to evaluate the broad areas on HER2 recognised by each antibody.
