# Supplementary material for: An IgE antibody targeting HER2 identified by clonal selection restricts breast cancer growth via immune-stimulating activities
Source: J Exp Clin Cancer Res. 2025 Feb 12;44:49. doi: 10.1186/s13046-025-03319-5 (PMC11818027; doi:10.1186/s13046-025-03319-5)
Supplement: Supplementary file 2 — Supplementary Material 2. Supplementary Fig. 2.pdf – Evaluation of IgE antibodies to interfere with the phosphorylation of HER2. SKBR3 were incubated in the presence of hEGF for 30 min prior addition of IgE antibodies at 5 µg/ml, resulting in varying levels of inhibition of phosphorylation of hHER2 (n = 4). Data presented as pHER2 normalised to the total HER2 measured for each condition. Data shown as mean ± SD. Source data are provided as a Source Data file. One-way ANOVA compared to isotype control; ****p ≤ 0.0001. [file 13046_2025_3319_MOESM2_ESM.pdf]

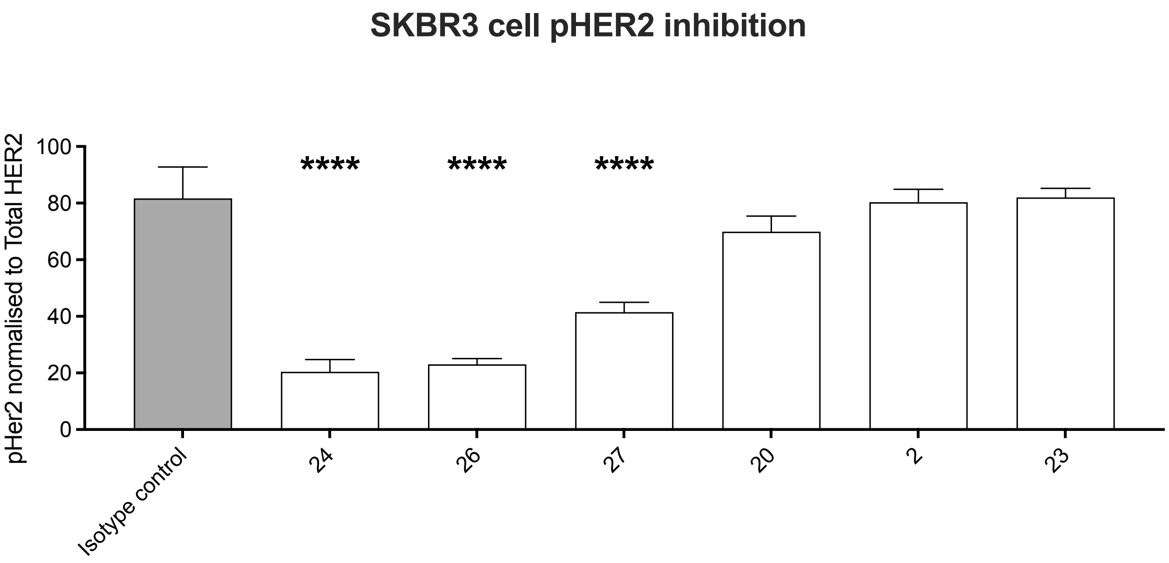

Supplementary Figure 2: Evaluation of IgE antibodies to interfere with the phosphorylation of HER2. SKBR3 were incubated in the presence of hEGF for 30 min prior addition of IgE antibodies at 5µg/ml, resulting in varying levels of inhibition of phosphorylation of hHER2 (n=4). Data presented as pHER2 normalised to the total HER2 measured for each condition. Data shown as mean ± SD. Source data are provided as a Source Data file. One-way ANOVA compared to isotype control; ****$p \leq 0.0001$.
