# Supplementary material for: An IgE antibody targeting HER2 identified by clonal selection restricts breast cancer growth via immune-stimulating activities
Source: J Exp Clin Cancer Res. 2025 Feb 12;44:49. doi: 10.1186/s13046-025-03319-5 (PMC11818027; doi:10.1186/s13046-025-03319-5)
Supplement: Supplementary file 3 — Supplementary Material 3. Supplementary Fig. 3.pdf – Measurements of affinity of human IgE antibodies for human HER2 and FcεRI. Surface Plasmon Resonance was conducted to evaluate IgE antibody affinity to human recombinant FcεRIα and human recombinant HER2. [file 13046_2025_3319_MOESM3_ESM.pdf]

3 Human IgEs

hFcεRIα

20 23 26

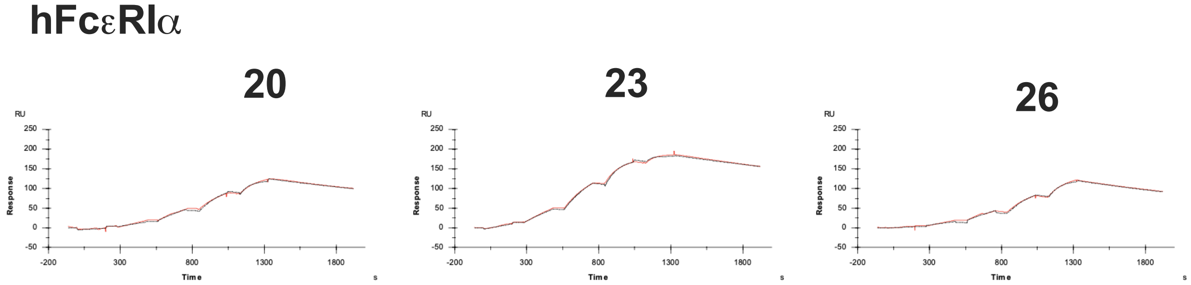

Antibody	ka (1/Ms)	kd (1/s)	K_D (M)
**20**	4.27E+05	3.84E-04	8.98E-10
**23**	9.44E+05	2.80E-04	2.96E-10
**26**	3.63E+05	4.59E-04	1.26E-09

hHER2

20 23 26

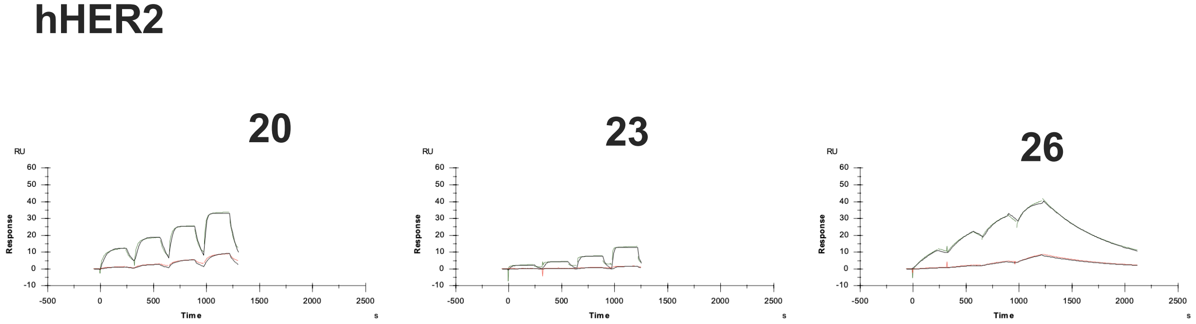

Antibody	ka (1/Ms)	kd (1/s)	K_D (M)
**20**	3.94E+05	1.34E-02	3.40E-08
**23**	4.57E+05	3.02E-02	6.61E-08
**26**	6.54E+04	1.50E-03	2.29E-08

Supplementary Figure 3: Measurements of affinity of human IgE antibodies for human HER2 and FcεRI. Surface Plasmon Resonance was conducted to evaluate IgE antibody affinity to human recombinant FcεRIα and human recombinant HER2.
