# Supplementary material for: An IgE antibody targeting HER2 identified by clonal selection restricts breast cancer growth via immune-stimulating activities
Source: J Exp Clin Cancer Res. 2025 Feb 12;44:49. doi: 10.1186/s13046-025-03319-5 (PMC11818027; doi:10.1186/s13046-025-03319-5)
Supplement: Supplementary file 4 — Supplementary Material 4. Supplementary Fig. 4.pdf – Evaluation of human and rat IgE antibody-dependent cellular phagocytosis (ADCP) of rat breast cancer cells. (A) Representative flow cytometric ADCC/ADCP assay plots: R1: total (CFSE stained) FITC + tumor cell population; R2: (CFSE stained) FITC + /APC + phagocytosed cells; R3: (CFSE stained) FITC + /DAPI + dead tumor cells. (B) Human IgEs 20, 23 and 26 (n = 4) and (C) rat IgE counterparts (n = 9) were evaluated for ability to trigger ADCP of rat HER2-expressing MTLn3 breast cancer cells by rat PMBC, measured by flow cytometry. Data shown as mean ± SD. Source data are provided as a Source Data file. One-way ANOVA showed no significant difference between the level of ADCP with controls or test IgE antibodies (B, C). [file 13046_2025_3319_MOESM4_ESM.pdf]

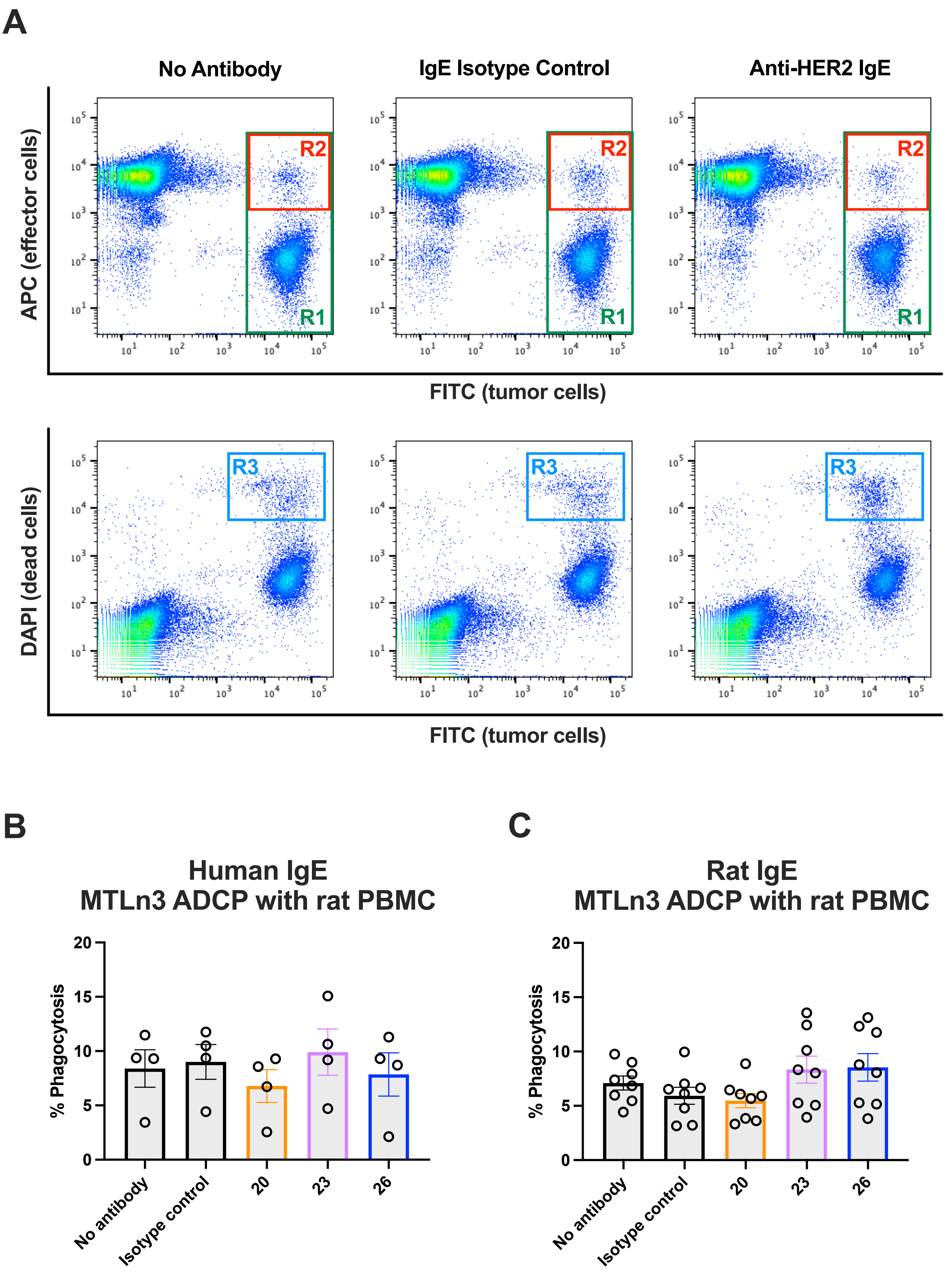

Supplementary Figure 4: Evaluation of human and rat IgE antibody-dependent cellular phagocytosis (ADCP) of rat breast cancer cells. Evaluation of human and rat IgE antibody-dependent cellular phagocytosis (ADCP) of rat breast cancer cells. (**A**) Representative flow cytometric ADCC/ADCP assay plots: R1: total (CFSE stained)

FITC+ tumor cell population; R2: (CFSE stained) FITC+/APC+ phagocytosed cells; R3: (CFSE stained) FITC+/DAPI+ dead tumor cells. (**B**) Human IgEs 20, 23 and 26 (n= 4) and (**C**) rat IgE counterparts (n=9) were evaluated for ability to trigger ADCP of rat HER2-expressing MTLn3 breast cancer cells by rat PMBC, measured by flow cytometry. Data shown as mean ± SD. Source data are provided as a Source Data file. One-way ANOVA showed no significant difference between the level of ADCP with controls or test IgE antibodies (**B**, **C**).
