# Supplementary material for: An IgE antibody targeting HER2 identified by clonal selection restricts breast cancer growth via immune-stimulating activities
Source: J Exp Clin Cancer Res. 2025 Feb 12;44:49. doi: 10.1186/s13046-025-03319-5 (PMC11818027; doi:10.1186/s13046-025-03319-5)
Supplement: Supplementary file 5 — Supplementary Material 5. Supplementary Fig. 5.pdf – Affinity measurements of rat IgE antibodies for human FcεRI and HER2. Surface Plasmon Resonance was conducted to evaluate rat IgE antibody affinity to human recombinant FcεRIα and human recombinant HER2. [file 13046_2025_3319_MOESM5_ESM.pdf]

3 Rat IgEs

hFcεRIα

20

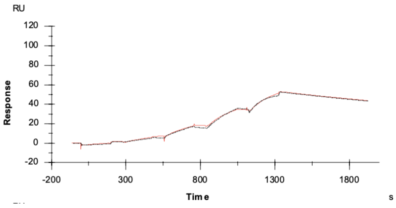

23

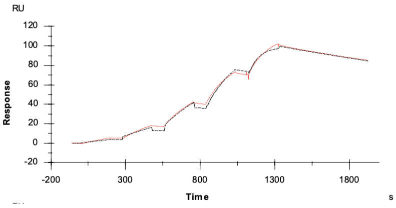

26

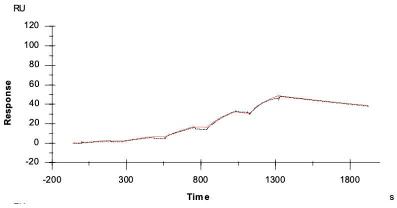

Antibody	ka (1/Ms)	kd (1/s)	K_D (M)
**20**	3.40E+05	3.29E-04	9.69E-10
**23**	4.36E+05	2.77E-04	6.36E-10
**26**	3.34E+05	3.29E-04	1.17E-09

hHER2

20

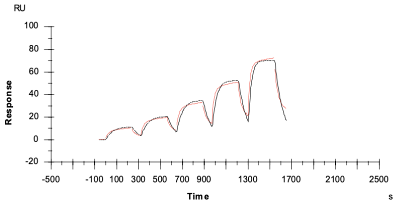

23

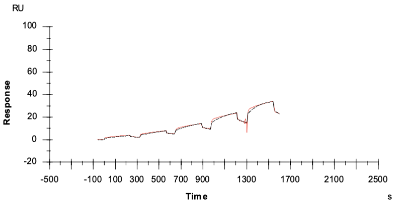

26

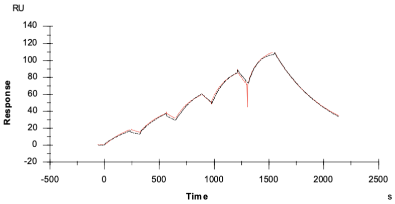

Antibody	ka (1/Ms)	kd (1/s)	K_D (M)
**20**	1.65E+05	1.35E-02	8.17E-08
**23**	3.93E+04	2.43E-03	6.19E-08
**26**	6.54E+04	1.99E-03	3.05E-08

Supplementary Figure 5: Affinity measurements of rat IgE antibodies for human FcεRI and HER2. Surface Plasmon Resonance was conducted to evaluate rat IgE antibody affinity to human recombinant FcεRIα and human recombinant HER2.
