# Supplementary material for: An IgE antibody targeting HER2 identified by clonal selection restricts breast cancer growth via immune-stimulating activities
Source: J Exp Clin Cancer Res. 2025 Feb 12;44:49. doi: 10.1186/s13046-025-03319-5 (PMC11818027; doi:10.1186/s13046-025-03319-5)
Supplement: Supplementary file 6 — Supplementary Material 6. Supplementary Fig. 6.pdf – Assessment of toxicity with IgE administration immunocompetent syngeneic rat model of HER2-expressing MTLn3 breast cancer in vivo. Summary of adverse events observed with intravenous administration of MTLn3 tumor bearing rats with either PBS (control), rat IgEs 20, 23 or 26, shown for each treatment condition and across different doses. Bottom right graph: animal weight measurements for each treatment group over time until termination. [file 13046_2025_3319_MOESM6_ESM.pdf]

Advserse events (AEs)

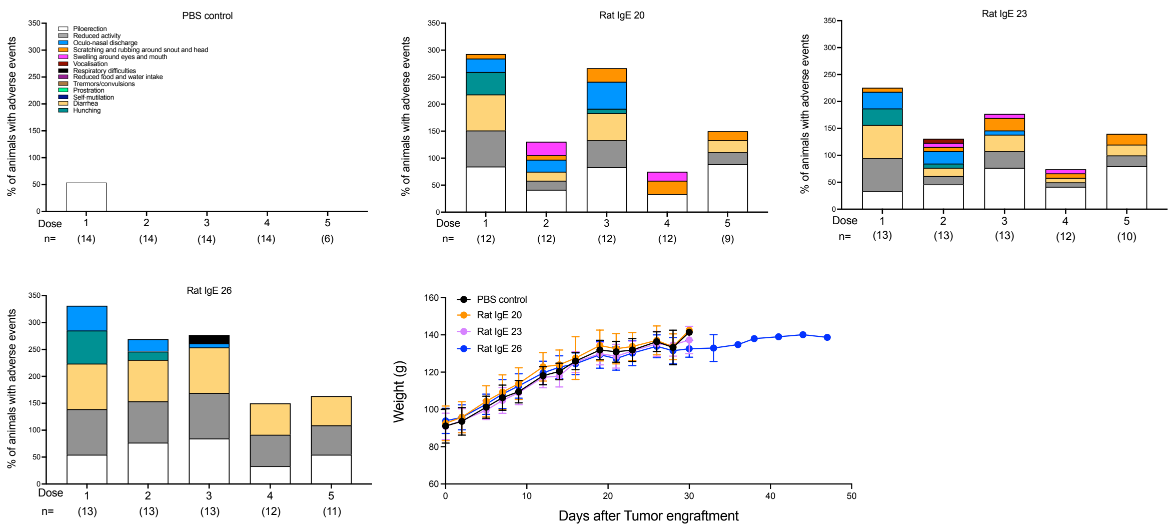

Supplementary Figure 6: Assessment of toxicity with IgE administration immunocompetent syngeneic rat model of HER2-expressing MTLn3 breast cancer *in vivo*. Summary of adverse events observed with intravenous administration of MTLn3 tumor bearing rats with either PBS (control), rat IgEs 20, 23 or 26, shown for each treatment condition and across different doses. Bottom right graph: animal weight measurements for each treatment group over time until termination.
