# Supplementary material for: An IgE antibody targeting HER2 identified by clonal selection restricts breast cancer growth via immune-stimulating activities
Source: J Exp Clin Cancer Res. 2025 Feb 12;44:49. doi: 10.1186/s13046-025-03319-5 (PMC11818027; doi:10.1186/s13046-025-03319-5)
Supplement: Supplementary file 7 — Supplementary Material 7. Supplementary Fig. 7.pdf – Transcriptomic analyses of tumor specimens from rats treated with anti-HER2 IgE. Deconvolution analyses revealed differential abundance of M2 macrophages, cytotoxic cells, and NK cells in tumors from animals treated with rat IgE 26. Welch’s t-test; ns not significant; *p ≤ 0.05. [file 13046_2025_3319_MOESM7_ESM.pdf]

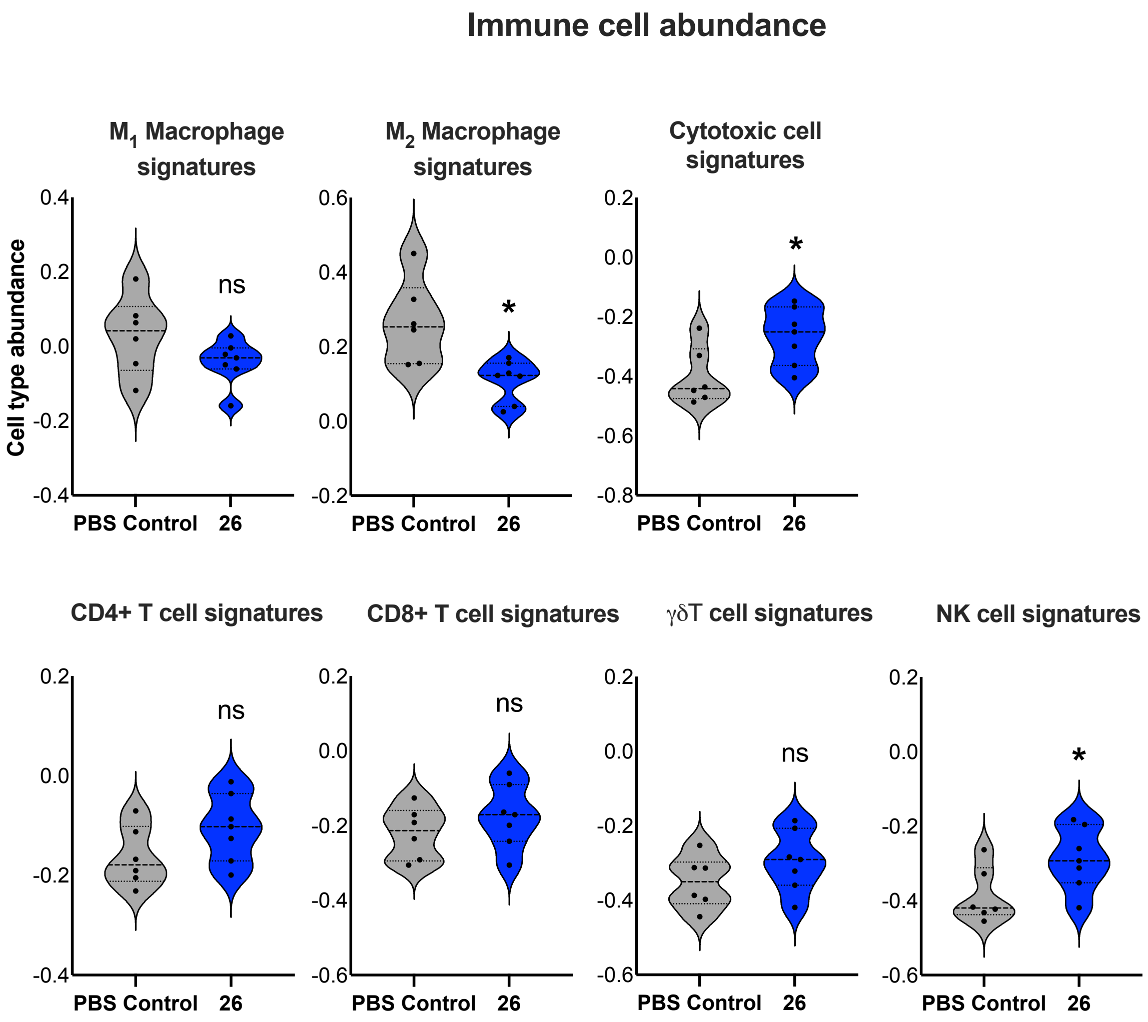

Supplementary Figure 7: Transcriptomic analyses of tumor specimens from rats treated with anti-HER2 IgE. Deconvolution analyses revealed differential abundance of M2 macrophages, cytotoxic cells, and NK cells in tumors from animals treated with rat IgE 26. Welch’s t-test; ns not significant; *p ≤ 0.05.
