# Supplementary material for: An IgE antibody targeting HER2 identified by clonal selection restricts breast cancer growth via immune-stimulating activities
Source: J Exp Clin Cancer Res. 2025 Feb 12;44:49. doi: 10.1186/s13046-025-03319-5 (PMC11818027; doi:10.1186/s13046-025-03319-5)
Supplement: Supplementary file 8 — Supplementary Material 8. Supplementary Fig. 8.pdf – Fab-mediated direct effects of rat IgE antibodies against MTLn3 cancer cells in vitro. The rat IgEs 20, 23 and 26 were evaluated for ability to affect different tumor cell mechanisms compared with IgE isotype control treatment in vitro. Antibody effects were measured on colony formation (A) and migration (B). Top: Bar graphs show colony formation and cellular migration measurements from 6 and 4 independent experiments, respectively. Bottom: Representative images of colony formation and migration. Data shown as mean ± SD. Source data are provided as a Source Data file. One-way ANOVA (A, B) *p ≤ 0.05; ***p ≤ 0.001. [file 13046_2025_3319_MOESM8_ESM.pdf]

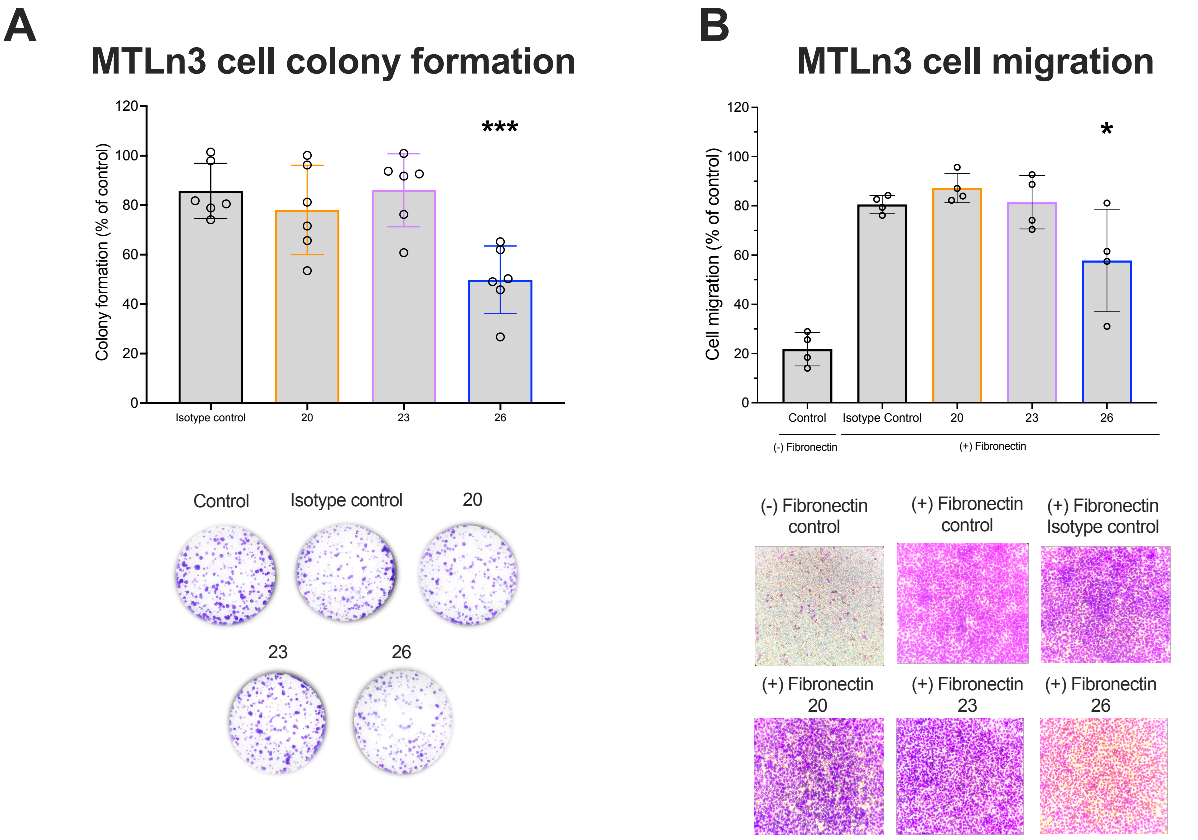


**Supplementary Figure 8: Fab-mediated direct effects of rat IgE antibodies against MTLn3 cancer cells *in vitro*.** The rat IgEs 20, 23 and 26 were evaluated for ability to affect different tumor cell mechanisms compared with IgE isotype control treatment *in vitro*. Antibody effects were measured on colony formation (A) and migration (B). Top: Bar graphs show colony formation and cellular migration measurements from 6 and 4 independent experiments, respectively. Bottom: Representative images of colony formation and migration. Data shown as mean ± SD. Source data are provided as a Source Data file. One-way ANOVA (A, B) *$p \leq 0.05$; ***$p \leq 0.001$.
