# Supplementary material for: An IgE antibody targeting HER2 identified by clonal selection restricts breast cancer growth via immune-stimulating activities
Source: J Exp Clin Cancer Res. 2025 Feb 12;44:49. doi: 10.1186/s13046-025-03319-5 (PMC11818027; doi:10.1186/s13046-025-03319-5)
Supplement: Supplementary file 9 — Supplementary Material 9. Supplementary Fig. 9.pdf – Confirmation of resistance to Fab-mediated direct effects of JIMT-1 human breast cancer cells. JIMT-1 human breast cancer cells show resistance to Fab-mediated functions of human IgE 26 in a ligand-independent cell viability in vitro assay (n = 4). Data shown as mean ± SD. Source data are provided as a Source Data file. Two-way ANOVA showed no significant difference between isotype control and V26 IgE at any concentration. [file 13046_2025_3319_MOESM9_ESM.pdf]

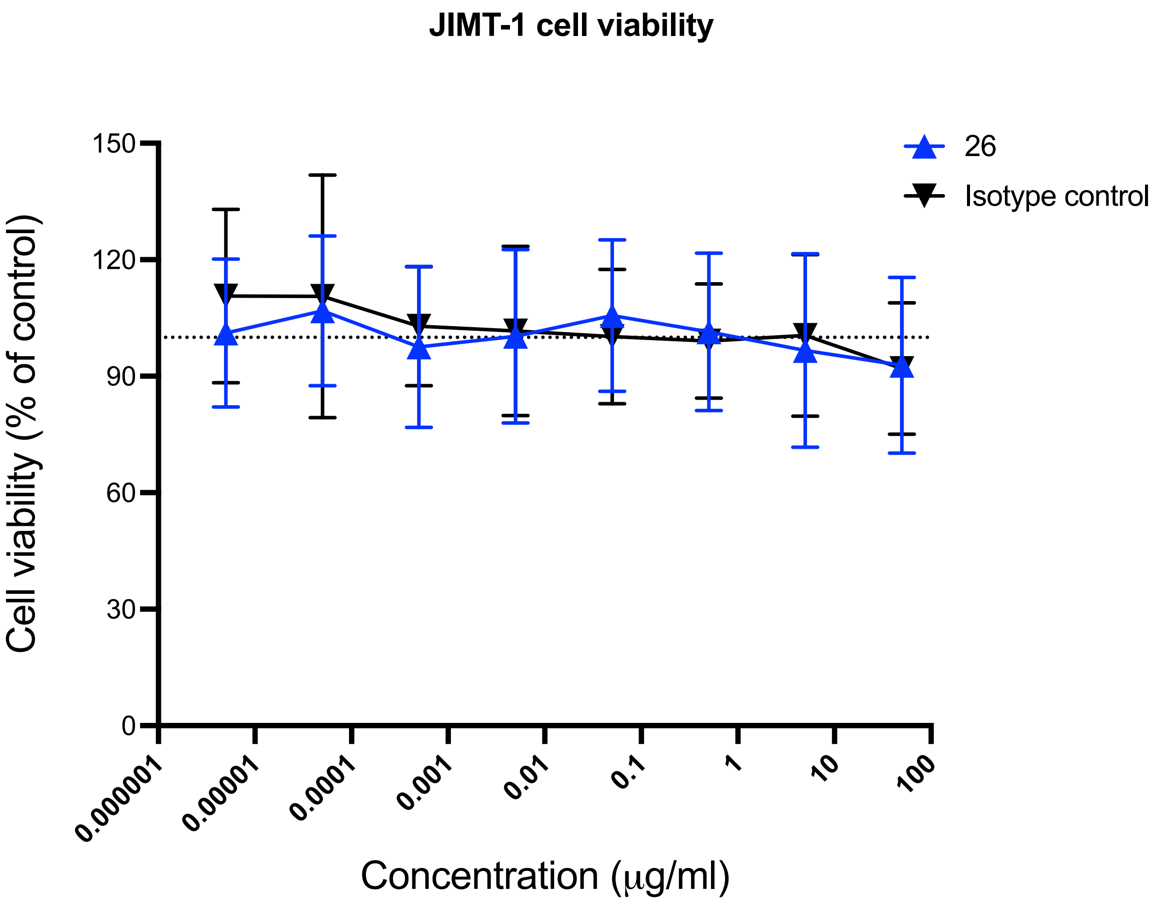

Supplementary Figure 9: Confirmation of resistance to Fab-mediated direct effects of JIMT-1 human breast cancer cells. JIMT-1 human breast cancer cells show resistance to Fab-mediated functions of human IgE 26 in a ligand-independent cell viability *in vitro* assay (n=4). Data shown as mean ± SD. Source data are provided as a Source Data file. Two-way ANOVA showed no significant difference between isotype control and V26 IgE at any concentration.
