# Supplementary material for: An IgE antibody targeting HER2 identified by clonal selection restricts breast cancer growth via immune-stimulating activities
Source: J Exp Clin Cancer Res. 2025 Feb 12;44:49. doi: 10.1186/s13046-025-03319-5 (PMC11818027; doi:10.1186/s13046-025-03319-5)
Supplement: Supplementary file 11 — Supplementary Material 11: Supplementary Table 2. Significantly differently-expressed genes (DEGs) in transcriptomic analyses of immune signatures within rat IgE 26-treated tumor specimens from immunocompetent syngeneic rat model of HER2-expressing MTLn3 breast cancer. [file 13046_2025_3319_MOESM11_ESM.docx]

**Supplementary Table 2** – Significantly differently-expressed genes (DEGs) in transcriptomic analyses of immune signatures within rat IgE 26-treated tumor specimens from immunocompetent syngeneic rat model of HER2-expressing MTLn3 breast cancer.

| Cell signature | Gene | p value (p adjusted) |
| --- | --- | --- |
| M1 | CCR1 | 0.0146 |
|  | CSF1 | 0.0100 |
|  | CSF1R | 0.0004 |
|  | CXCL10 | 0.0100 |
|  | CXCL11 | 0.0065 |
|  | CXCL9 | 0.0308 |
|  | CYBB | 0.0174 |
|  | HCK | 0.0412 |
|  | LILRB1 | 0.0290 |
|  | MMP19 | 0.0000 |
|  | SIGLEC1 | 0.0014 |
|  | TFEC | 0.0006 |
|  | TNFAIP6 | 0.0005 |
| M2 | CFP | 0.0292 |
|  | CLEC10A | 0.0037 |
|  | CLEC4A | 0.0032 |
|  | HCK | 0.0412 |
|  | MMP19 | 0.0000 |
|  | MS4A6A | 0.0004 |
|  | TFEC | 0.0006 |
| Cytotoxic cells | CTSW | 0.0108 |
|  | KLRB1 | 0.0082 |
|  | KLRD1 | 0.0939 |
|  | KLRK1 | 0.0378 |
| CD4+ T cells | CD27 | 0.0355 |
|  | CD6 | 0.0348 |
|  | CD96 | 0.0051 |
|  | CTSW | 0.0108 |
|  | DPEP2 | 0.0020 |
|  | FCN1 | 0.0001 |
|  | GPR183 | 0.0096 |
|  | ITK | 0.0123 |
|  | KLRB1 | 0.0082 |
|  | TNFRSF4 | 0.0443 |
|  | TRAT1 | 0.0300 |
| CD8+ T cells | CCR5 | 0.0053 |
|  | CD160 | 0.0258 |
|  | CD27 | 0.0355 |
|  | CD300A | 0.0167 |
|  | CD96 | 0.0051 |
|  | CHST12 | 0.0036 |
|  | CTSW | 0.0108 |
|  | CYTH4 | 0.0345 |
|  | FASLG | 0.0180 |
|  | ITK | 0.0123 |
|  | KLRB1 | 0.0082 |
|  | KLRK1 | 0.0378 |
|  | LAG3 | 0.0270 |
|  | MMP19 | 5.75E-06 |
| γδ T cells | CCR5 | 0.0053 |
|  | CD160 | 0.0258 |
|  | CD300A | 0.0167 |
|  | CD96 | 0.0051 |
|  | CHST12 | 0.0036 |
|  | FASLG | 0.0180 |
|  | ITK | 0.0123 |
|  | KLRB1 | 0.0082 |
|  | LAG3 | 0.0270 |
| NK cells | CCR5 | 0.0053 |
|  | CD160 | 0.0258 |
|  | CTSW | 0.0108 |
|  | FASLG | 0.0180 |
|  | KLRB1 | 0.0082 |
|  | KLRK1 | 0.0378 |
|  | LAG3 | 0.0270 |
