# Supplementary material for: An IgE antibody targeting HER2 identified by clonal selection restricts breast cancer growth via immune-stimulating activities
Source: J Exp Clin Cancer Res. 2025 Feb 12;44:49. doi: 10.1186/s13046-025-03319-5 (PMC11818027; doi:10.1186/s13046-025-03319-5)
Supplement: Supplementary file 12 — Supplementary Material 12: Supplementary Table 3. Significantly differently-expressed genes (DEGs) in transcriptomic analyses of signalling pathways within rat IgE 26-treated tumor specimens from immunocompetent syngeneic rat model of HER2-expressing MTLn3 breast cancer. [file 13046_2025_3319_MOESM12_ESM.docx]

**Supplementary Table 3** – Significantly differently-expressed genes (DEGs) in transcriptomic analyses of signalling pathways within rat IgE 26-treated tumor specimens from immunocompetent syngeneic rat model of HER2-expressing MTLn3 breast cancer.

|  | Signalling pathway | Gene | p value (p adjusted) |
| --- | --- | --- | --- |
| FcεRI | FcεRI-mediated MAPK activation | LAT | 0.04751 |
|  |  | IGLV6-57 | 0.03463 |
|  |  | IGHV4-34 | 0.00310 |
|  |  | IGHV4-39 | 0.00426 |
|  |  | IGHV4-59 | 0.00426 |
|  | FcεRI-mediated NF-κβ activation | PSMB10 | 0.02136 |
|  |  | PSMB9 | 0.01519 |
|  |  | IGLV6-57 | 0.03463 |
|  |  | IGHV4-34 | 0.00310 |
|  |  | IGHV4-39 | 0.00426 |
|  |  | IGHV4-59 | 0.00426 |
|  | FcεRI-mediated Ca^2+^ mobilisation | LAT | 0.04751 |
|  |  | ITK | 0.01235 |
|  |  | IGLV6-57 | 0.03463 |
|  |  | IGHV4-34 | 0.00310 |
|  |  | IGHV4-39 | 0.00426 |
|  |  | IGHV4-59 | 0.00426 |
| Tumor proliferation | Signaling by receptor tyrosine kinases | THBS4 | 1.51E-06 |
|  |  | FGF7 | 0.0002 |
|  |  | FGF10 | 0.0196 |
|  |  | FLRT2 | 0.0003 |
|  |  | VEGFD | 0.0006 |
|  |  | NGF | 0.0012 |
|  |  | COL3A1 | 0.0001 |
|  |  | ARC | 0.0089 |
|  |  | COL5A1 | 0.0009 |
|  |  | GRB10 | 0.0001 |
|  |  | LAMA2 | 0.0005 |
|  |  | PDGFRA | 0.0001 |
|  |  | FGF16 | 0.0439 |
|  |  | CXCL12 | 0.0024 |
|  |  | CDH5 | 0.0001 |
|  |  | COL5A2 | 0.0005 |
|  |  | VEGFC | 0.0017 |
|  |  | COL1A1 | 0.0004 |
|  |  | FN1 | 0.0004 |
|  |  | ADAM12 | 0.0371 |
|  |  | LAMA4 | 0.0006 |
|  |  | COL5A3 | 0.0023 |
|  |  | COL6A6 | 0.0356 |
|  |  | SPARC | 0.0002 |
|  |  | PCSK5 | 0.0006 |
|  |  | DLG4 | 0.0029 |
|  |  | FLT4 | 0.0007 |
|  |  | ITGB3 | 0.0220 |
|  |  | PCSK6 | 0.0086 |
|  |  | LAMB1 | 0.0001 |
|  |  | TGFBR3 | 0.0022 |
|  |  | DNM3 | 0.0124 |
|  |  | IGF1 | 0.0054 |
|  |  | CAV1 | 0.0029 |
|  |  | COL11A1 | 0.0057 |
|  |  | COL4A1 | 0.0006 |
|  |  | COL4A2 | 0.0084 |
|  |  | PRKCZ | 0.0133 |
|  | Gα(q) signaling events | NTSR1 | 0.0020 |
|  |  | LPAR1 | 0.0007 |
|  |  | PTGFR | 0.0087 |
|  |  | AGTR1 | 0.0007 |
|  |  | BDKRB1 | 0.0076 |
|  |  | RGS4 | 0.0007 |
|  |  | LPAR3 | 0.0345 |
|  |  | PLCB1 | 0.0013 |
|  |  | MGLL | 0.0184 |
|  |  | LPAR4 | 0.0145 |
|  |  | GNG4 | 0.0002 |
|  |  | AVPR1A | 0.0020 |
|  |  | CYSLTR1 | 0.0338 |
|  |  | HRH1 | 0.0189 |
|  |  | RGS5 | 0.0076 |
|  |  | KALRN | 0.0141 |
|  |  | HTR2A | 0.0222 |
|  |  | GPR4 | 0.0002 |
|  |  | GNB4 | 0.0150 |
|  |  | BDKRB2 | 0.0247 |
|  |  | RPS6KA2 | 0.0317 |
|  |  | EDNRA | 0.0258 |
|  |  | GNB5 | 0.0261 |
|  |  | BTK | 0.0471 |
| Tumor migration | Integrin cell surface interactions | FBN1 | 7.00E-05 |
|  |  | COL3A1 | 0.0001 |
|  |  | COL5A1 | 0.0009 |
|  |  | TNC | 0.0018 |
|  |  | COL5A2 | 0.0005 |
|  |  | LUM | 0.0010 |
|  |  | COL1A1 | 0.0004 |
|  |  | FN1 | 0.0004 |
|  |  | JAM3 | 0.0002 |
|  |  | COL5A3 | 0.0023 |
|  |  | ICAM2 | 0.0001 |
|  |  | COL6A6 | 0.0356 |
|  |  | ITGB3 | 0.0220 |
|  |  | COL8A1 | 0.0003 |
|  |  | COL4A1 | 0.0006 |
|  |  | COL4A2 | 0.0084 |
|  |  | KDR | 0.0042 |
|  |  | IBSP | 0.0406 |
|  |  | ITGA9 | 0.0169 |
|  |  | COL6A1 | 0.0015 |
|  |  | JAM2 | 0.0024 |
|  |  | COL6A2 | 0.0009 |
|  |  | VTN | 0.0261 |
|  |  | ITGA11 | 0.0157 |
|  |  | ITGAD | 0.0309 |
|  |  | COL4A4 | 0.0180 |
|  |  | ITGB2 | 0.0111 |
|  |  | VWF | 0.0103 |
|  |  | ITGA5 | 0.0451 |
|  |  | COL16A1 | 0.0435 |
|  | MET promotes cell mobility | COL3A1 | 0.0001 |
|  |  | COL5A1 | 0.0009 |
|  |  | LAMA2 | 0.0005 |
|  |  | COL5A2 | 0.0005 |
|  |  | COL1A1 | 0.0004 |
|  |  | FN1 | 0.0004 |
|  |  | LAMA4 | 0.0006 |
|  |  | COL5A3 | 0.0023 |
|  |  | LAMB1 | 0.0001 |
|  |  | COL11A1 | 0.0057 |
|  |  | COL27A1 | 0.0261 |
|  |  | ITGB1 | 0.0048 |
|  |  | GRB2 | 0.0414 |
|  | L1CAM interactions | GAP43 | 0.02423 |
|  |  | DCX | 0.02729 |
|  |  | ANK2 | 0.02896 |
|  |  | DLG4 | 0.00287 |
|  |  | EPHB2 | 0.03272 |
|  |  | NCAM1 | 0.00797 |
|  |  | ITGB3 | 0.02201 |
|  |  | LAMB1 | 0.00013 |
|  |  | CNTN2 | 0.03656 |
|  |  | DNM3 | 0.01237 |
|  |  | ITGA9 | 0.01690 |
|  |  | NRP1 | 0.00466 |
|  |  | RPS6KA2 | 0.03166 |
|  |  | TUBB3 | 0.00433 |
|  |  | AP2A2 | 0.00599 |
|  |  | ITGA5 | 0.04514 |
|  |  | ITGB1 | 0.00479 |
|  |  | MSN | 0.01378 |
| Tumor invasion | Degradation of extracelular matrix | ELN | 0.0003 |
|  |  | ADAMTS5 | 0.0009 |
|  |  | FBN1 | 7.00E-05 |
|  |  | COL3A1 | 0.0001 |
|  |  | COL5A1 | 0.0009 |
|  |  | TLL1 | 0.0002 |
|  |  | FBN2 | 0.0021 |
|  |  | COL5A2 | 0.0005 |
|  |  | DCN | 0.0003 |
|  |  | MMP19 | 5.75E-06 |
|  |  | NID1 | 0.0004 |
|  |  | COL1A1 | 0.0004 |
|  |  | FN1 | 0.0004 |
|  |  | MMP2 | 0.0016 |
|  |  | COL5A3 | 0.0023 |
|  |  | MMP16 | 0.0330 |
|  |  | COL6A6 | 0.0356 |
|  |  | COL15A1 | 0.0266 |
|  |  | COL8A1 | 0.0003 |
|  |  | LAMB1 | 0.0001 |
|  |  | ADAMTS16 | 0.0200 |
|  |  | COL12A1 | 0.0168 |
|  |  | COL11A1 | 0.0057 |
|  |  | COL4A1 | 0.0006 |
|  |  | COL4A2 | 0.0084 |
|  |  | ADAMTS4 | 0.0005 |
|  |  | MMP17 | 0.0343 |
|  |  | COL6A1 | 0.0015 |
|  |  | CTSK | 0.0029 |
|  |  | ADAMTS1 | 0.0057 |
|  |  | COL6A2 | 0.0009 |
|  |  | COL4A4 | 0.0180 |
|  |  | MMP14 | 0.0263 |
|  |  | TIMP2 | 0.0009 |
|  | GPCR ligand binding | CRHR1 | 0.0005 |
|  |  | NTSR1 | 0.0020 |
|  |  | CD55 | 1.76E-05 |
|  |  | WNT2 | 0.0263 |
|  |  | ACKR3 | 3.43E-05 |
|  |  | CCR1 | 0.0146 |
|  |  | APLNR | 0.0001 |
|  |  | PENK | 0.0117 |
|  |  | LPAR1 | 0.0007 |
|  |  | RXFP1 | 0.0382 |
|  |  | CXCL12 | 0.0024 |
|  |  | PTGFR | 0.0087 |
|  |  | AGTR1 | 0.0007 |
|  |  | BDKRB1 | 0.0076 |
|  |  | HTR1B | 0.0471 |
|  |  | LPAR3 | 0.0345 |
|  |  | GIP | 0.0373 |
|  |  | LPAR4 | 0.0145 |
|  |  | GNG4 | 0.0002 |
|  |  | AVPR1A | 0.0020 |
|  |  | CYSLTR1 | 0.0338 |
|  |  | CCL7 | 2.08E-05 |
|  |  | S1PR3 | 0.0017 |
|  |  | HRH1 | 0.0189 |
|  |  | CCL2 | 0.0001 |
|  |  | C3AR1 | 0.0125 |
|  |  | S1PR1 | 0.0005 |
|  |  | CRHR2 | 0.0418 |
| Angiogenesis | ECM proteoglycans | COL3A1 | 0.0001 |
|  |  | COL5A1 | 0.0009 |
|  |  | LAMA2 | 0.0005 |
|  |  | TNC | 0.0018 |
|  |  | COL5A2 | 0.0005 |
|  |  | VCAN | 0.0008 |
|  |  | LUM | 0.0010 |
|  |  | DCN | 0.0003 |
|  |  | COL1A1 | 0.0004 |
|  |  | FN1 | 0.0004 |
|  |  | BGN | 0.0002 |
|  |  | LAMA4 | 0.0006 |
|  |  | COL5A3 | 0.0023 |
|  |  | COL6A6 | 0.0356 |
|  |  | SPARC | 0.0002 |
|  |  | NCAM1 | 0.0080 |
|  |  | ITGB3 | 0.0220 |
|  |  | LAMB1 | 0.0001 |
|  |  | COL4A1 | 0.0006 |
|  |  | COL4A2 | 0.0084 |
|  |  | IBSP | 0.0406 |
|  |  | ITGA9 | 0.0169 |
|  |  | COL6A1 | 0.0015 |
|  |  | SERPINE1 | 0.0001 |
|  |  | COL6A2 | 0.0009 |
|  |  | VTN | 0.0261 |
|  |  | COL4A4 | 0.0180 |
|  |  | TGFB2 | 0.0004 |
|  | Collagen formation | SERPINH1 | 1.98E-05 |
|  |  | COLGALT2 | 0.0081 |
|  |  | COL3A1 | 0.0001 |
|  |  | PLOD2 | 0.0005 |
|  |  | LOX | 0.0001 |
|  |  | COL5A1 | 0.0009 |
|  |  | TLL1 | 0.0002 |
|  |  | COL5A2 | 0.0005 |
|  |  | COL1A1 | 0.0004 |
|  |  | PXDN | 0.0010 |
|  |  | ADAMTS2 | 0.0004 |
|  |  | ADAMTS14 | 0.0024 |
|  |  | COL5A3 | 0.0023 |
|  |  | COL6A6 | 0.0356 |
|  |  | PCOLCE | 0.0003 |
|  |  | LOXL2 | 0.0052 |
|  |  | COL15A1 | 0.0266 |
|  |  | COL8A1 | 0.0003 |
|  |  | LOXL1 | 0.0003 |
|  |  | COL12A1 | 0.0168 |
|  |  | COL11A1 | 0.0057 |
|  |  | COL4A1 | 0.0006 |
|  |  | COL4A2 | 0.0084 |
|  |  | LOXL3 | 0.0062 |
|  |  | COL6A1 | 0.0015 |
|  |  | COL27A1 | 0.0261 |
|  |  | P4HA3 | 0.0136 |
|  |  | COL6A2 | 0.0009 |
|  |  | COL4A4 | 0.0180 |
|  |  | P3H3 | 0.0173 |
|  |  | P3H1 | 0.0040 |
|  |  | P4HA1 | 0.0026 |
|  |  | PLOD1 | 0.0106 |
|  |  | P4HA2 | 0.0265 |
|  |  | PCOLCE2 | 0.0232 |
|  |  | COL16A1 | 0.0435 |
|  |  | PLOD3 | 0.0005 |
|  | Signaling by PDGF | THBS4 | 1.51E-06 |
|  |  | COL3A1 | 0.0001 |
|  |  | COL5A1 | 0.0009 |
|  |  | PDGFRA | 0.0001 |
|  |  | COL5A2 | 0.0005 |
|  |  | COL5A3 | 0.0023 |
|  |  | COL6A6 | 0.0356 |
|  |  | COL4A1 | 0.0006 |
|  |  | COL4A2 | 0.0084 |
|  |  | COL6A1 | 0.0015 |
|  |  | COL6A2 | 0.0009 |
|  |  | PDGFD | 0.0071 |
|  |  | COL4A4 | 0.0180 |
|  |  | THBS2 | 0.0105 |
|  |  | PDGFRB | 0.0229 |
|  |  | PLAT | 0.0023 |
|  |  | PDGFB | 0.0021 |
