# Supplementary material for: An IgE antibody targeting HER2 identified by clonal selection restricts breast cancer growth via immune-stimulating activities
Source: J Exp Clin Cancer Res. 2025 Feb 12;44:49. doi: 10.1186/s13046-025-03319-5 (PMC11818027; doi:10.1186/s13046-025-03319-5)
Supplement: Supplementary file 13 — Supplementary Material 13: Supplementary Table 4. Statistical analysis of rat IgE 26 dose and scheduling study in immunocompetent syngeneic rat model of HER2-expressing MTLn3 breast cancer. [file 13046_2025_3319_MOESM13_ESM.docx]

**Supplementary Table 4** – Statistical analysis of rat IgE 26 dose and scheduling study in immunocompetent syngeneic rat model of HER2-expressing MTLn3 breast cancer.

| Days | PBS vs rat IgE 26 20mg/kg BIW | PBS vs rat IgE 26 20mg/kg QW | PBS vs rat IgE 26 10mg/kg BIW | PBS vs rat IgE 26 10mg/kg QW |
| --- | --- | --- | --- | --- |
| 12 | ns | ns | ns | ns |
| 14 | ns | ns | ns | ns |
| 16 | ns | ns | ns | ns |
| 19 | ns | ns | ns | ns |
| 21 | ns | ns | ns | ns |
| 23 | ns | ** | ns | * |
| 26 | **** | **** | **** | **** |
