# Supplementary material for: An IgE antibody targeting HER2 identified by clonal selection restricts breast cancer growth via immune-stimulating activities
Source: J Exp Clin Cancer Res. 2025 Feb 12;44:49. doi: 10.1186/s13046-025-03319-5 (PMC11818027; doi:10.1186/s13046-025-03319-5)
Supplement: Supplementary file 14 — Supplementary Material 14: Supplementary Table 5. Statistical analysis of rat IgE 26 compared to isotype control IgE in immunocompetent syngeneic rat model of HER2-expressing MTLn3 breast cancer. [file 13046_2025_3319_MOESM14_ESM.docx]

**Supplementary Table 5** - Statistical analysis of rat IgE 26 compared to isotype control IgE in immunocompetent syngeneic rat model of HER2-expressing MTLn3 breast cancer.

| Days | PBS vs rat IgE 26  7mg/kg BIW | PBS vs isotype control 7mg/kg BIW | Isotype control vs rat IgE 26 7mg/kg BIW |
| --- | --- | --- | --- |
| 11 | ns | ns | ns |
| 14 | ns | ns | ns |
| 16 | ns | ns | ns |
| 18 | ns | ns | ns |
| 21 | ns | ns | ns |
| 22 | ns | ns | ns |
| 23 | ns | ns | ns |
| 25 | **** | ns | **** |
