# Supplementary material for: An IgE antibody targeting HER2 identified by clonal selection restricts breast cancer growth via immune-stimulating activities
Source: J Exp Clin Cancer Res. 2025 Feb 12;44:49. doi: 10.1186/s13046-025-03319-5 (PMC11818027; doi:10.1186/s13046-025-03319-5)
Supplement: Supplementary file 16 — Supplementary Material 16: Supplementary Table 7. Statistical analysis of human IgE 26 dose and scheduling study in a human breast cancer xenograft model (QW). [file 13046_2025_3319_MOESM16_ESM.docx]

**Supplementary Table 7** – Statistical analysis of human IgE 26 dose and scheduling study in a human breast cancer xenograft model (QW).

| Days | PBS vs human IgE 26 20mg/kg QW | PBS vs human IgE 26 10mg/kg QW | PBS vs human IgE 26 2mg/kg QW | Human IgE 26 2mg/kg vs human IgE 26 10mg/kg QW | Human IgE 26 2mg/kg vs human IgE 26 20mg/kg QW | Human IgE 26 10mg/kg vs human IgE 26 20mg/kg QW |
| --- | --- | --- | --- | --- | --- | --- |
| 1 | ns | ns | ns | ns | ns | ns |
| 2 | ns | ns | ns | ns | ns | ns |
| 4 | ns | ns | ns | ns | ns | ns |
| 7 | ns | ns | ns | ns | ns | ns |
| 9 | ns | ns | ns | ns | ns | ns |
| 11 | ns | ns | ns | ns | ns | ns |
| 14 | * | ns | ns | ns | ns | ns |
| 16 | *** | ** | ns | ns | ns | ns |
| 18 | **** | ** | ns | ns | ns | ns |
| 21 | **** | *** | ns | ns | * | ns |
| 23 | **** | **** | * | ns | * | ns |
| 25 | **** | **** | ** | ns | * | ns |
| 28 | **** | **** | ** | ns | ** | ns |
| 29 | **** | **** | ** | ns | ** | ns |
