# Supplementary material for: An IgE antibody targeting HER2 identified by clonal selection restricts breast cancer growth via immune-stimulating activities
Source: J Exp Clin Cancer Res. 2025 Feb 12;44:49. doi: 10.1186/s13046-025-03319-5 (PMC11818027; doi:10.1186/s13046-025-03319-5)
Supplement: Supplementary file 18 — Supplementary Material 18: Supplementary Table 9. Statistical analysis of human IgE 26 compared to isotype control IgE in a human breast cancer xenograft model. [file 13046_2025_3319_MOESM18_ESM.docx]

**Supplementary Table 9** - Statistical analysis of human IgE 26 compared to isotype control IgE in a human breast cancer xenograft model.

| Days | PBS vs isotype control IgE | Isotype control vs human IgE 26  20mg/kg BIW |
| --- | --- | --- |
| 1 | ns | ns |
| 3 | ns | ns |
| 6 | ns | ns |
| 8 | ns | ns |
| 10 | ns | ns |
| 13 | ns | ns |
| 15 | ns | * |
| 17 | ns | ** |
| 20 | ns | ** |
| 22 | ns | **** |
| 24 | ns | **** |
| 27 | ns | **** |
| 29 | ns | **** |
