# Supplementary material for: An IgE antibody targeting HER2 identified by clonal selection restricts breast cancer growth via immune-stimulating activities
Source: J Exp Clin Cancer Res. 2025 Feb 12;44:49. doi: 10.1186/s13046-025-03319-5 (PMC11818027; doi:10.1186/s13046-025-03319-5)
Supplement: Supplementary file 20 — Supplementary Material 20: Supplementary Table 11. Statistical analysis of human IgE 26 -PBMCs in a human breast cancer xenograft model. [file 13046_2025_3319_MOESM20_ESM.docx]

**Supplementary Table 11** - Statistical analysis of human IgE 26 -PBMCs in a human breast cancer xenograft model.

| Days | PBS vs human IgE 26 20mg/kg BIW  -PBMCs |
| --- | --- |
| 1 | ns |
| 2 | ns |
| 4 | ns |
| 7 | ns |
| 9 | ns |
| 11 | ns |
| 14 | ns |
| 16 | ns |
| 18 | ns |
| 21 | ns |
| 23 | ns |
| 25 | ns |
| 28 | ns |
| 29 | ns |
